# Reelin Exerts Structural, Biochemical and Transcriptional Regulation Over Presynaptic and Postsynaptic Elements in the Adult Hippocampus

**DOI:** 10.3389/fncel.2016.00138

**Published:** 2016-05-30

**Authors:** Carles Bosch, Ashraf Muhaisen, Lluís Pujadas, Eduardo Soriano, Albert Martínez

**Affiliations:** ^1^Department of Cell Biology, Physiology and Immunology, Faculty of Biology, University of BarcelonaBarcelona, Spain; ^2^Centro de Investigación Biomédica en Red sobre Enfermedades Neurodegenerativas (CIBERNED), MadridSpain; ^3^Vall d’Hebron Institut de RecercaBarcelona, Spain; ^4^Institute of Neurosciences, University of BarcelonaBarcelona, Spain; ^5^Institució Catalana de Recerca i Estudis Avançats AcademiaBarcelona, Spain

**Keywords:** dendritic spines, spine apparatus, NMDA receptors, axon terminal, electron microscopy

## Abstract

Reelin regulates neuronal positioning and synaptogenesis in the developing brain, and adult brain plasticity. Here we used transgenic mice overexpressing Reelin (Reelin-OE mice) to perform a comprehensive dissection of the effects of this protein on the structural and biochemical features of dendritic spines and axon terminals in the adult hippocampus. Electron microscopy (EM) revealed both higher density of synapses and structural complexity of both pre- and postsynaptic elements in transgenic mice than in WT mice. Dendritic spines had larger spine apparatuses, which correlated with a redistribution of Synaptopodin. Most of the changes observed in Reelin-OE mice were reversible after blockade of transgene expression, thus supporting the specificity of the observed phenotypes. Western blot and transcriptional analyses did not show major changes in the expression of pre- or postsynaptic proteins, including SNARE proteins, glutamate receptors, and scaffolding and signaling proteins. However, EM immunogold assays revealed that the NMDA receptor subunits NR2a and NR2b, and p-Cofilin showed a redistribution from synaptic to extrasynaptic pools. Taken together with previous studies, the present results suggest that Reelin regulates the structural and biochemical properties of adult hippocampal synapses by increasing their density and morphological complexity and by modifying the distribution and trafficking of major glutamatergic components.

## Introduction

Reelin, a large secreted glycoprotein of the extracellular matrix, controls neuronal migration and brain development (D’Arcangelo et al., 1995; Alcantara et al., 1998; Rice and Curran, 2001; Soriano and Del Rio, 2005; Cooper, 2008; D’Arcangelo, 2014). Reelin binds to lipoprotein family receptors apolipoprotein E receptor 2 (ApoER2) and very-low-density lipoprotein receptor (VLDL; D’Arcangelo et al., 1999; Hiesberger et al., 1999), and induces the phosphorylation of the adaptor protein mDab1 (Howell et al., 1999; Ballif et al., 2004). The Reelin cascade therefore includes several signaling pathways, including distinct members of the Src Kinase family (Arnaud et al., 2003),Erk1/2 (Simo et al., 2007; Lee et al., 2014) and AKT/Gsk3 (Beffert et al., 2002; Gonzalez-Billault et al., 2005). The duration of the Reelin signaling is regulated by ubiquitination and degradation of p-mDab1, triggered by the Cul5 cascade (Feng et al., 2007; Simo et al., 2010; Simo and Cooper, 2013).

In the adult cerebral cortex, Reelin is expressed mainly by γ-amino-butyric acid (GABA) interneurons (Alcantara et al., 1998; Pesold et al., 1998). Reelin promotes the maintenance of synaptic connectivity by modulating synaptic plasticity (Beffert et al., 2005; Bender et al., 2010; Pujadas et al., 2010) and by regulating the composition and traffic of NMDA and AMPA receptor subunits (Chen et al., 2005; Qiu et al., 2006b; Groc et al., 2007; Qiu and Weeber, 2007). Moreover, *in vitro* and *in vivo* studies have revealed that both mDab1 mutant mice and Reeler mice show a reduction in spine density, thereby supporting the notion that Reelin is involved in synaptic development and/or maturation (Niu et al., 2004, 2008; Ventruti et al., 2011). Similarly, local *in vivo* injections of Reelin in wild-type mice promote an increase in spine density and synapse formation (Rogers et al., 2011, 2013). In parallel, transgenic mice overexpressing Reelin (Reelin-OE) show hypertrophy of dendritic spines in the adult hippocampus (Pujadas et al., 2010). It is known that dendritic spine morphology is linked to synaptic efficacy (Bourne and Harris, 2008; Colgan and Yasuda, 2014). For example, spines are enlarged after long-term potentiation (LTP) – induced stimulation (Matsuzaki et al., 2004; Yang et al., 2008), and both the spine head volume and the number of AMPA-type glutamate receptors increase synaptic strength (Takumi et al., 1999; Matsuzaki et al., 2001). Taken together, these findings suggest that Reelin modulates synaptic efficacy not only by regulating the density of dendritic spines but also by controlling dendritic spine and synaptic architecture. Finally, recent studies point to the participation of Reelin in Alzheimer’s disease and in the synaptopathies associated with this condition (Knuesel, 2010; Pujadas et al., 2014; Lane-Donovan et al., 2015).

To understand the role of this protein in adult neural function, there is a need for a better knowledge of the fine regulation of the structural and molecular characteristics of dendritic spines and synapses, specifically in the adult brain *in vivo*. Here we take advantage of transgenic mice that overexpress Reelin exclusively in late postnatal and adult forebrain, with no impact on neural migration (Pujadas et al., 2010) to determine the effects of Reelin overexpression on the structural and molecular phenotype of dendritic spines and synaptic terminals in the hippocampus. Our data provide the first evidence that Reelin modulates the structural and molecular properties of adult synapses by altering the synaptic distribution of glutamate receptors and associated proteins rather than by controlling the protein and gene expression levels of the same.

## Materials and Methods

### Animals

Transgenic Reelin-OE mice of either sex (TgRln) used in this study have been previously described (Pujadas et al., 2010). This line overexpresses Reelin ectopically under the calcium–calmodulin-dependent kinase IIa promoter (pCaMKIIα) through a tTA transactivator. Feeding for 1 week with doxycycline-containing feed (Bio-Serv, 200 mg per kg) successfully blocks the transgene expression (Pujadas et al., 2010). Mice were housed in groups (2–6 mice per cage) and maintained in a 12-h light-dark cycle with access to food and water *ad libitum*. All procedures were performed in accordance to the protocols approved by local ethics committee and in compliance with the European guidelines for humane treatment of laboratory animals.

### Synaptosome Extracts and Western Blots

Hippocampal synaptosome-enriched protein extracts were obtained following a protocol described elsewhere (Niu et al., 2008). Briefly, adult mice were sacrificed by decapitation, brains were removed from the skull, and hippocampi were dissected in ice-cold dissection buffer and pooled (four hippocampi per sample). Hippocampi were homogenized in 1.5 ml of SP buffer with an Eppendorf tissue grinder. A 150 μl aliquot of homogenate was kept for validation purposes. The homogenate was centrifuged twice at 800*g* for 5 min at 4°C in a F45-24-11 rotor on an Eppendorf^®^ 5415R centrifuge, and pellets were discarded. The cleaned supernatant was then centrifuged at 5769*g* for 11 min at 4°C in a TLA-55 rotor on a Beckmann OPTIMA TLX ultracentrifuge. The supernatant was then carefully removed, and the pellet was resuspended in 250 μl of lysis buffer.

Western blots against various proteins of interest were performed using the following antibodies: Actin (mouse monoclonal “mAb” clone C4; 1:100000; Millipore), Synapsin 2 (rabbit polyclonal “poly”; 1:500; Stressgen Bioreagents), SNAP25 (mouse mAb clone SMI-81; 1:750; Becton-Dickinson), Synaptopodin (rabbit poly; 1:750; Synaptic Systems), NR2a (rabbit poly; 1:500; Millipore), NR2b (rabbit poly; 1:500; Millipore), p-Cofilin on serine 3 (rabbit poly; 1/100; Santa Cruz), NR1 (mouse mAb clone 54.1; 1/1000; BD Biosciences), GluR1 (rabbit mAb clone C3T; 1/500; Millipore), GluR2/3 (rabbit poly; 1/1000; Chemicon), postsynaptic density (PSD)-95 (mouse mAb clone 7E3-1B8; 1:1000; Millipore), CaMKII (mouse mAb clone 6G9; 1/2000; Affinity Bioreagents), LIMK-1 (rabbit poly; 1/100; Santa Cruz), phospho-LIMK1 (Thr508)/LIMK2 (Thr505; rabbit poly; 1/500; Cell Signaling Technology) and Cofilin (rabbit poly; 1/500; Millipore).

### Immunohistochemistry

Five-month-old Reelin transgenic mice (*n* = 3), control littermates (*n* = 3), and Reelin transgenic mice treated with doxycycline (DOX) for 7 days (*n* = 3) were anesthetized and perfused with 4% paraformaldehyde in 0.1 M Phosphate buffer. Brains were removed, post-fixed overnight in the same solution, cryoprotected, and frozen. They were then coronally sectioned at 30 μm, distributed into 10 series, and maintained at -20°C in cryoprotectant solution (PB 0.1 M, sucrose 30% and glycerol-30% ethylene glycol). For the immunodetection of Synaptopodin, sections were blocked for 2 h at RT with PBS containing 10% of normal goat serum (NGS) and 0.2% of gelatin and then incubated with rabbit anti-Synaptopodin (1:500, Synaptic Systems) overnight at 4°C with PBS–5% NGS. Next, they were incubated with goat anti-rabbit fluorochrome-labeled secondary antibodies (1:700, Molecular Probes), mounted in Mowiol, and stored at -20°C. Sections were viewed in a Leica SP2 confocal scanning laser microscope. The acquisition of confocal stacks of 3–5 dorsal hippocampi was achieved in a Leica SP2 microscope. Images were taken at a 20× magnification with a z-spacing of 2 μm. To allow comparison between all the animal groups, sections were immunolabeled in bulk and imaged in identical conditions. Acquisition *x*- and *y*-resolution was set at 1.46 μm/px. From each stack, the intensity of two to three confocal slices was z-projected. Regions of interest (ROIs) were defined across various layers (5 to 10 ROIs per layer), avoiding histological artifacts such as vessels or cell nuclei from which gray values were recorded. Intensities were normalized to average slice intensity to retrieve a local contrast index comparable across acquisitions.

### Gene Expression Microarray Analysis of Reelin-OE Mice

The differential expression of genes directly associated with the term “Synapse” (GO:0045202) was examined from data collected in the analysis of the genome-wide expression pattern of whole hippocampus. Briefly, mouse hippocampi (*n* = 5–7 mice per group) were dissected out and immediately frozen in liquid nitrogen. mRNAs were extracted using a Trizol (Invitrogen)-based protocol, quantified in Qubit Fluorometer (Life Technologies), and subjected to quality control using a Bioanalyzer (Agilent). cDNAs were synthesized and amplified by Ovation System (NuGEN) and hybridized to GeneChip Mouse Genome 430 2.0 array (Affymetrix) at the *Functional Genomics Core Facility* (IRB Barcelona). GeneChips were scanned in a GeneChip Scanner 3000 (Affymetrix). Full analysis of data was performed at the *Biostatistics/Bioinformatics Unit* (IRB Barcelona), as previously described (Rossell et al., 2008). Of the 396 genes directly associated with GO:0045202, 360 gene probe sets with 871 independent probes are represented in the GeneChip (details in **Supplementary Table S1**). In addition, probes corresponding to genes Limk1/Limk2/Cfl1/Cfl2 were also analyzed (details in **Supplementary Table S2**). Thresholds for considering differential expression were set at *probability* of differential expression >0.95, and absolute fold change >1.25.

### Electron Microscopy (EM) and Post-embedding Immunogold Immunohistochemistry

Adult Reelin transgenic mice (*n* = 3), control littermates (*n* = 3), and Reelin transgenic mice treated with DOX for 7 days (*n* = 3) were perfused with 2% glutaraldehyde-2% paraformaldehyde in 0.12 M phosphate buffer. After post-fixation in the same solution overnight, tissue slices were transferred to 2% osmium tetroxide, stained with 2% uranyl acetate, dehydrated, and embedded with araldite. Ultrathin sections were then obtained from at least two araldite blocks per hippocampus, and stained with lead citrate. Electron micrographs (at 25,000×) of each hippocampal layer were randomly taken, and the area, circularity index and phenotype of spines receiving at least one synaptic contact were determined (*n* = 112–161 spines for each layer and group). Moreover, the density of dendritic spines showing spine apparatus (SA; *n* = 30–70 electron micrographs analyzed per layer and group) and the area of this organelle (*n* = 44–83 spine apparatuses) were calculated. In this case, a SA was considered to be measured if at least two tubules of smooth endoplasmic reticulum were detected in close apposition (Deller et al., 2006). The analyzed images corresponded to randomly selected fields within the neuropil in the layer of interest. We excluded the sampling of serial, consecutive sections, to avoid the possibility that any given item could be counted twice. Areas of spine apparatuses were measured using ImageJ software (Schindelin et al., 2012). Finally, we analyzed the area and circularity index of axon terminals and the length of their synaptic contacts (*n* = 92–171 synaptic terminals for each layer and group).

For post-embedding immunostaining analysis, adult Reelin transgenic mice (*n* = 2), control littermates (*n* = 2) and Reelin transgenic mice treated with DOX for 7 days (*n* = 2) were perfused with 0.1% glutaraldehyde-4% paraformaldehyde in 0.12 M phosphate buffer and processed. After removal, brains were cryoprotected gradually in sucrose and cryofixed by immersion in liquid propane. Freeze substitution was performed at -90°C for 3 days in an “Automatic Freeze Substitution System” (AFS, Leica), using methanol containing 0.5% uranyl acetate as substitution medium. Brains were infiltrated in Lowicryl HM20 at -50°C and then polymerized with UV lamps. Ultrathin sections were obtained from one block per hippocampus, collected and processed for post-embedding immunostaining. Samples were incubated with either rabbit anti-NR2a (1:5, Millipore Bioscience Research Reagents), rabbit anti-NR2b (1:5, Millipore Bioscience Research Reagents), or rabbit anti-p-Cofilin (1:5, Cell Sign Tech) antiserum. Sections were then incubated with goat anti-rabbit 18-nm gold-tagged antibody (1:20; British Biocell International). These sections were then counterstained with uranyl acetate and lead citrate. Electron micrographs (30,000×) were obtained in the stratum radiatum (SR) of the hippocampus CA1 region, and the number of dots per dendritic spine was counted in those spines that contained at least two gold particles (*n* = 20–35 spines per group). We classified each in-spine dot as belonging to one out of three compartments based on the features that lied within a 20 nm radius from the dot: synaptic (PSD), extrasynaptic (plasma membrane) or cytoplasmic (intracellular; dots not meeting the criteria for either synaptic or extrasynaptic).

### Statistical Analyses

In all analyses, animal genotype was blind to the experimenter. The number of animals used in each experiment is detailed above. Statistical analysis was performed using non-parametric one-way ANOVA (Kruskal–Wallis test) followed by Dunns’s post-test.

## Results

### Reelin Overexpression Modulates the Structural Complexity of Presynaptic Terminals

**FIGURE 1:**
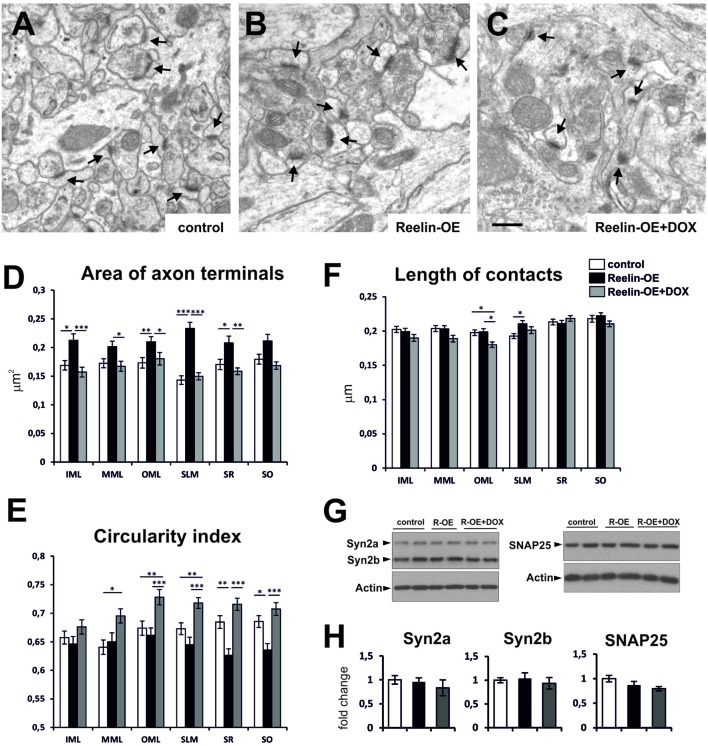
**Fine structure and molecular phenotype of axon terminals in Reelin-OE mice. (A–C)** Electron micrographs illustrating axon terminals establishing synaptic contacts (arrows) in the inner molecular layer of control, Reelin-OE, and DOX-treated Reelin-OE mice. **(D–F)** Histograms showing area **(D)**, circularity index **(E)**, and length of synaptic contact **(F)** of axon terminals in different hippocampal layers in control, Reelin-OE, and DOX-treated Reelin-OE mice (mean ± SEM; *p < 0.05, **p < 0.01, ***p < 0.001; ANOVA test). **(G)** Western-blot analysis of a and b isoforms of Synapsin 2, and SNAP25 in synaptosomal fractions of the hippocampus. **(H)** Quantification of Synapsin 2a, Synapsin 2b, and SNAP-25 values from triplicate experiments. Values are normalized to the levels of actin and expressed as percentage of control mice (mean ± SEM, ANOVA test). IML, inner molecular layer; MML, medial molecular layer, OML, outer molecular layer; SLM, stratum lacunosum-moleculare; SO, stratum oriens; SR, stratum radiatum. Scale bar in **(A–C)**, 0.5 μm.

We recently reported that Reelin overexpression induces a higher density of synaptic contacts in the adult hippocampus (Pujadas et al., 2010). Here we first further analyzed the ultrastructural and molecular phenotypes of synaptic elements in the different layers of the hippocampus of control and Reelin-OE mice. We also examined Reelin-OE mice in which the transgene had been switched off 1 week earlier by DOX treatment. Regarding presynaptic elements, axon terminals in Reelin-OE mice were larger (16–62%) than those in controls in all hippocampal layers (**Figures 1A–D**). The greatest increase was found in the stratum lacunosum-moleculare (SLM). The circularity index—used as a measure of the morphological complexity of axon terminals—tended to be lower in Reelin-OE mice than in controls, although this reduction was significant only in the SR and stratum oriens (SO; 7–8%; **Figure 1E**). Both the axonal bouton area and the circularity index returned to normal values 1 week after transgene inactivation, thus reinforcing the specificity of the observed phenotypes (**Figures 1D,E**). In two hippocampal layers, however, the circularity index in the Reelin-OE-DOX group surpassed the control values, a feature often observed for other parameters in both the present study and previously (Pujadas et al., 2010). Finally, we measured the length of the synaptic contacts, observing a significant increase in the SLM of Reelin-OE mice (**Figure 1F**).

To elucidate whether the molecular presynaptic machinery was also altered in Reelin-OE mice, we performed Western blot analyses against distinct synaptic proteins in lysates from hippocampal synaptosomal fractions. The protein expression levels for a number of presynaptic markers, including Synapsin 2a and 2b, and SNAP-25 (**Figures 1G,H**), Synaptophysin, VAMP-2, and Syntaxin 1 (data not shown), remained unchanged across the experimental groups. These results suggest that Reelin overexpression modulates the structural features of hippocampal axon terminals but does not substantially alter the protein levels of the presynaptic machinery linked to exocytosis and neurotransmitter release.

### Reelin Overexpression Regulates Postsynaptic Dendritic Spine Morphology

**FIGURE 2:**
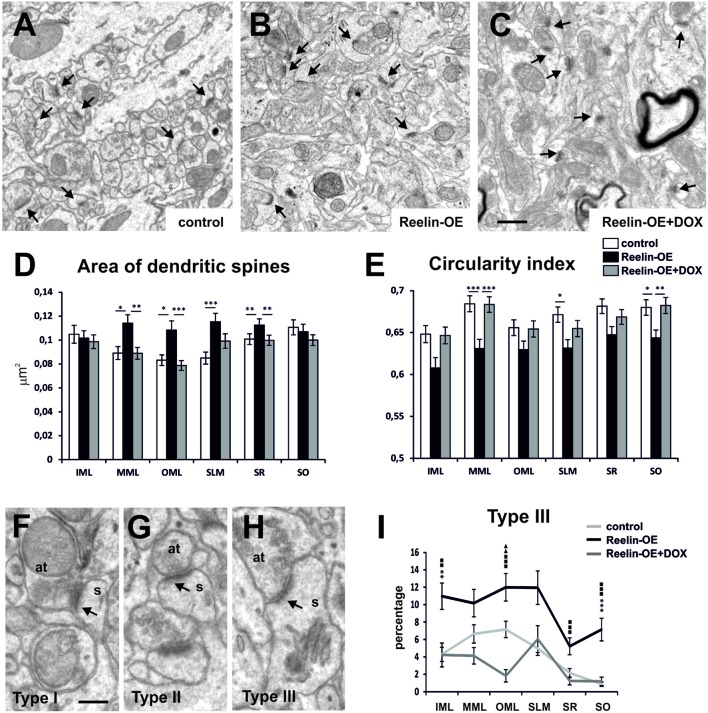
**Fine structure of dendritic spines in Reelin-OE mice. (A–C)** Electron micrographs illustrating dendritic spines receiving synaptic inputs (arrows) in the inner molecular layer of control, Reelin-OE, and DOX-treated Reelin-OE mice. **(D,E)** Histograms showing area **(D)** and circularity index **(E)** of dendritic spines in different hippocampal layers in control, Reelin-OE, and DOX-treated Reelin-OE mice (mean ± SEM; *p < 0.05, **p < 0.01, ***p < 0.001; ANOVA test). **(F–H)** Electron micrographs showing three types of defined dendritic spines depending on PSD curvature (see text): type I **(F)**, type II **(G)**, and type III **(H)**. **(I)** Histogram illustrating the percentage of type III dendritic spines in all the hippocampal layers in control, Reelin-OE, and DOX-treated Reelin-OE mice (mean ± SEM; *p < 0.05, **p < 0.01, ***p < 0.001 for control vs. Reelin-OE mice; squares for Reelin-OE vs. Reelin-OE+DOX; triangles for control vs. Reelin-OE+DOX mice; ANOVA test). IML, inner molecular layer; MML, medial molecular layer, OML, outer molecular layer; SLM, stratum lacunosum-moleculare; SO, stratum oriens; SP, stratum pyramidale. Scale bars, **(A–C)** = 0.5 μm; **(F–H)** = 200 nm.

By reconstructing hippocampal dendritic segments, we recently found that dendritic spines were hypertrophied in the SR of Reelin-OE mice (Pujadas et al., 2010). To substantiate these findings, we conducted a detailed fine structural analysis on postsynaptic dendritic spines in the different hippocampal layers (**Figure 2**). The surface of dendritic spines was larger in most hippocampal layers in Reelin-OE mice than in control mice (**Figures 2A,B**). Differences were greater in the layers receiving entorhinal input, i.e., the medial molecular layer (MML), the outer molecular layer (OML) and the SLM (27–35% of increase; **Figure 2D**). Furthermore, in Reelin-OE mice treated with DOX dendritic spine surfaces returned to control values (**Figures 2C,D**). The circularity index of dendritic spine heads, a parameter that reflects their complexity, was lower in all the hippocampal layers of Reelin-OE mice than in their littermate controls (**Figure 2E**). Again, Reelin-OE-DOX animals exhibited spine circularity index values close to those detected in control mice (**Figure 2E**).

Dendritic spine shapes are associated with spine function and plasticity (Bourne and Harris, 2008). We thus analyzed the phenotype of hippocampal dendritic spines in Reelin-OE mice. The most widely used nomenclature to distinguish the morphological diversity of dendritic spines is based on the relative size (and shape) of the spine head and neck (Harris et al., 1992; Bourne and Harris, 2008). Following these criteria, dendritic spines are classified as thin, stubby, or mushroom type. However, this grouping requires fully imaged dendritic spines. Since our analysis was based on single section EM micrographs, we implemented a new spine classification criteria based on the curvature of the postsynaptic density (PSD), a parameter suitable for the analysis of sectioned spines. On the basis of the curvature of the PSD and postsynaptic region, we distinguished the following types of spines: type I, showing a convex PSD (**Figure 2F**); type II, showing a straight or weakly concave PSD (**Figure 2G**); and type III, showing a PSD with a sufficiently concave curvature to fit an area of the pre-synaptic bouton large enough to accommodate an entire synaptic vesicle (**Figure 2H**). These synaptic types are likely to correspond to the classical thin, stubby, and mushroom types, respectively (Marrone and Petit, 2002; Marrone et al., 2004; Medvedev et al., 2010a). Following this classification, we observed that while some layers showed significant differences in the percentage of types I and II (**Supplementary Figure S1**), the number of type III dendritic spines was higher in all the hippocampal layers of Reelin-OE mice (from 1.5 to 8.2-fold, see **Figure 2I**), compared to their control littermates. These spine phenotypes were largely reverted by a 1-week treatment with DOX (**Figure 2I**). All together, these findings indicate that Reelin increases the structural complexity of pre- and postsynaptic elements and shifts a proportion of spines toward a type III phenotype, whose higher complexity recalls that of mushroom spines.

### Reelin Modulates the Complexity of Spine Apparatus in a Lamina-Specific Manner

**FIGURE 3:**
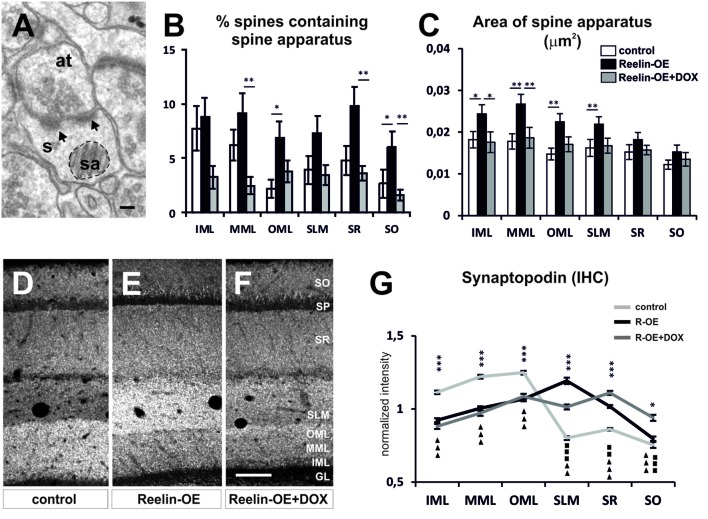
**Spine apparatus and Synaptopodin in dendritic spines of Reelin-OE mice. (A)** Electron micrograph of dendritic spines (s) containing a spine apparatus (sa) and receiving two synaptic contacts (arrows) from an axon terminal (at). Dashed-line circle represents an example of the area of spine apparatus used to perform analysis in **(C)**. **(B,C)** Histograms illustrating the percentage of dendritic spines containing spine apparatus **(B)** and the area of these organelles **(C)** in distinct hippocampal layers of control, Reelin-OE, and DOX-treated Reelin-OE mice (mean ± SEM; *p < 0.05, **p < 0.01; ANOVA test). **(D–F)** Immunolabeling for Synaptopodin in hippocampal sections of control, Reelin-OE, and DOX-treated Reelin-OE mice. **(G)** Quantitative determination of immunofluorescence signals in control, Reelin-OE, and Reelin-OE+DOX mice. Diagram shows continuous linear profiles of fluorescence intensities in vertical stripes of hippocampal sections from the stratum oriens to the granule layer for Synaptopodin. The intensity of fluorescence is represented in gray levels (mean ± SEM; *p < 0.05, **p < 0.01, ***p < 0.001 for control vs. Reelin-OE mice; squares for Reelin-OE vs. Reelin-OE+DOX; triangles for control vs. Reelin-OE+DOX mice; ANOVA test). GL, granular layer; IHC, immunohistochemistry; IML, inner molecular layer; MML, medial molecular layer; OML, outer molecular layer; SLM, stratum lacunosum-moleculare; SO, stratum oriens; SP, stratum pyramidale; WB, western blot. Scale bars, **(A)** = 100 nm; **(F,G)** = 100 μm.

The SA (**Figure 3A**), a specialized form of endoplasmic reticulum comprising several stacks (Spacek and Harris, 1997), has been implicated in dendritic spine function and plasticity as it regulates the synthesis and trafficking of glutamate receptors, among other functions (Jedlicka et al., 2008; Segal et al., 2010). Interestingly, we found an increase in the percentage of spines containing SA in all hippocampal layers of Reelin-OE mice, this increase being statistically significant in the OML (**Figure 3B**). Moreover, analysis of the surface occupied by SAs (e.g., **Figure 3A**) revealed that these organelles were significantly larger in Reelin-OE mice in the layers receiving entorhinal input, i.e., the MML, OML, and SLM (**Figure 3C**). Both quantitative measurements reverted to control values when Reelin-OE mice were treated with DOX (**Figures 3B,C**).

An essential component of the SA is the actin-associated protein Synaptopodin, which is also involved in synaptic plasticity (Mundel et al., 1997; Jedlicka et al., 2008; Segal et al., 2010; Zhang et al., 2013). Synaptopodin mRNA is expressed by both hippocampal pyramidal neurons and granule cells (Mundel et al., 1997), where it shows a lamina-specific distribution (Deller et al., 2000; Roth et al., 2001; Bas Orth et al., 2005). To determine whether Reelin overexpression altered Synaptopodin expression in the hippocampus, we performed immunohistochemical analyses and Western blot assays in the synaptosomal fractions. No significant differences were found in the overall level of protein expression among genotypes (**Supplementary Figure S2**). However, immunohistochemical analyses showed layer-specific alterations in the distribution of Synaptopodin in Reelin-OE mice (**Figures 3D–F**). Thus, in agreement with earlier studies (Deller et al., 2000; Bas Orth et al., 2005), control mice showed a highest expression of Synaptopodin in the molecular layer of the dentate gyrus (DG), with diffuse staining in the CA1 region (**Figures 3D,G**). In contrast, Reelin-OE mice showed a marked increase in Synaptopodin expression in the SLM (**Figures 3E,G**) and decreased expression in the DG. These differences were partially reversed in Reelin-OE mice treated with DOX (**Figures 3F,G**). Taken together, these results indicate that Reelin overexpression leads to a higher complexity of the SA and to a redistribution of Synaptopodin expression, being concentrated in the SLM, without altering the overall hippocampal expression levels of this protein.

### Reelin Regulates the Synaptic Distribution of NMDA Receptors

**FIGURE 4:**
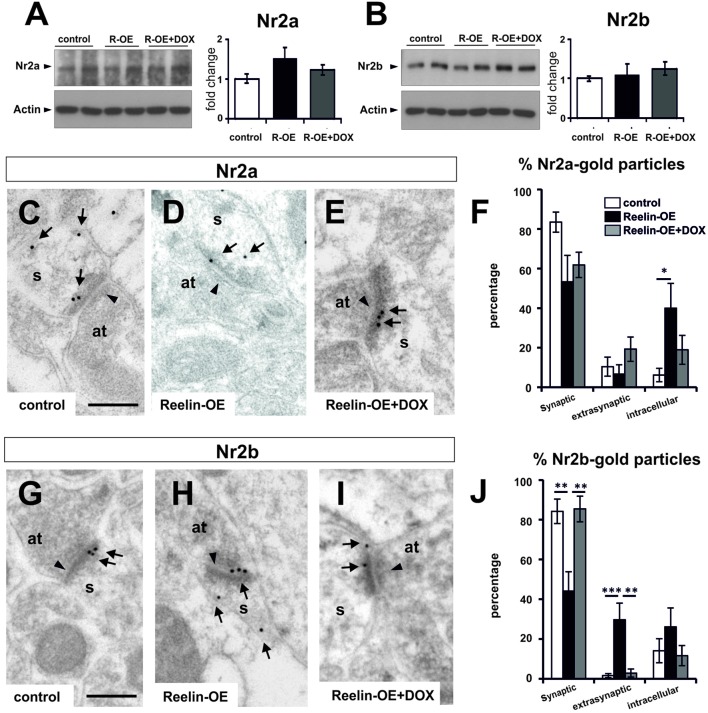
**Expression of NMDA receptor subunits NR2a and NR2b in Reelin-OE mice. (A)** Left, immunoblot analysis of NR2a in control, Reelin-OE, and DOX-treated Reelin-OE mice. Right, histogram showing densitometric analysis (*n* = 4 animals per group) by fold change in the three groups of mice (mean ± SEM; ANOVA test). **(B)** Left, immunoblot analysis of NR2b in control, Reelin-OE, and DOX-treated Reelin-OE mice. Right, histogram showing densitometric analysis (*n* = 3 animals per group) by fold change in the three groups of mice (mean ± SEM; ANOVA test). **(C–E)** Examples of immunogold labeling against NR2a in dendritic spines (s) receiving a synaptic contact from an axon terminal (at) in the SR of control **(C)**, Reelin-OE **(D)**, and DOX-treated Reelin-OE **(E)** mice. **(F)** Histogram illustrating the proportion of NR2a-gold particles in the synaptic contact, the extrasynaptic membrane and the intracellular domain of dendritic spines in the SR of Reelin-OE and DOX-treated Reelin-OE mice and their control littermates (mean ± SEM; *p < 0.05; ANOVA test). **(G–I)** Examples of immunogold labeling against NR2b in dendritic spines (s) receiving a synaptic contact from an axon terminal (at) in the SR of control **(G)**, Reelin-OE **(H)**, and DOX-treated Reelin-OE **(I)** mice. **(J)** Histogram illustrating the proportion of NR2b-gold particles in the synaptic contact, the extrasynaptic membrane and the intracellular domain of dendritic spines in the SR of Reelin-OE and DOX-treated Reelin-OE mice and their control littermates (mean ± SEM; **p < 0.01, ***p < 0.001; ANOVA test). In **(C–E,G–I)**, note synaptic contacts pointed by arrowheads and immunolabeling dots labeled by arrows. Scale bars, 250 nm.

**FIGURE 5:**
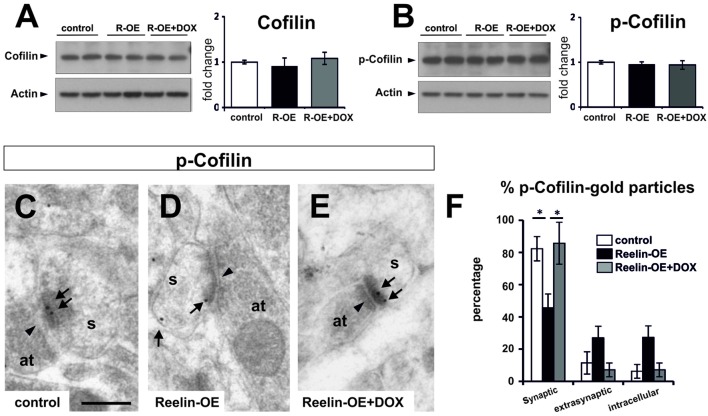
**Expression of Cofilin and p-Cofilin in Reelin-OE mice. (A)** Left, immunoblot analysis of Cofilin in control, Reelin-OE, and DOX-treated Reelin-OE mice. Right, histogram showing densitometric analysis (*n* = 3 animals per group) by fold change in the three groups of mice (mean ± SEM; ANOVA test). **(B)** Left, immunoblot analysis of p-Cofilin in control, Reelin-OE, and DOX-treated Reelin-OE mice. Right, histogram showing densitometric analysis (*n* = 3 animals per group) by fold change in the three groups of mice (mean ± SEM; ANOVA test). **(C–E)** Examples of immunogold labeling against p-Cofilin in dendritic spines (s) receiving a synaptic contact from an axon terminal (at) in the SR of control **(C)**, Reelin-OE **(D)**, and DOX-treated Reelin-OE **(E)** mice. Note synaptic contacts indicated by arrowheads and immunolabeling dots by arrows. Scale bar, 250nm. **(F)** Histogram illustrating the proportion of dots in the synaptic contact, the extrasynaptic membrane and the intracellular domain of dendritic spines in the SR of Reelin-OE and DOX-treated Reelin-OE mice and their control littermates (mean ± SEM; *p < 0.05; ANOVA test).

To study whether the molecular composition of postsynaptic elements is affected by Reelin-overexpression in the hippocampus, we performed Western blot assays on synaptosomal fractions. Thus, we analyzed the protein levels of several glutamate receptor subunits (GluR1, GluR2/3, GluN1, NR2a, and NR2b), scaffolding proteins (PSD-95), and downstream effector components (CaMKII, Cofilin, p-Cofilin, LIMK1, and p-LIMK1/LIMK2) (**Supplementary Figure S3**; **Figures 4A,B** and **5A,B**). Noteworthy, none of these proteins showed altered expression levels in purified synaptosomal fractions.

Given that Reelin expression has been linked to the surface mobility of NMDA receptors *in vitro* (Groc et al., 2007; Qiu and Weeber, 2007), we next addressed whether this protein influences the distribution of these receptor subunits *in vivo*. For this purpose, we performed immunogold EM staining against the NR2a and NR2b subunits, and the distribution of these subunits in the SR was plotted. The distribution of NR2a subunits was altered in Reelin-OE mice, compared to controls (**Figures 4C–E**). Thus, while 83% of NR2a receptors were found in postsynaptic densities in control spines, Reelin-OE mice showed a marked decrease in this location, concomitant with a higher number of intracellular (40%) receptor subunits (**Figure 4F**). Immunogold analyses of the NR2b subunits showed a similar altered distribution of synaptic versus intracellular subunits in Reelin-OE mice; moreover, Reelin-overexpression lead to marked increase in extrasynaptic NR2b subunits (**Figures 4G–J**). The effects of Reelin overexpression on NR2a/b synaptic/extrasynaptic distribution were largely reversed after arresting transgene expression with DOX (**Figures 4E,F,I,J**).

Cofilin, an F-actin severing protein, promotes the stabilization of mature dendritic spines. It is preferentially found in postsynaptic densities (Chai et al., 2009; Shi et al., 2009; Rust et al., 2010), and its phosphorylation has been proposed to be regulated by Reelin (Chai et al., 2009; Leemhuis and Bock, 2011). We thus tested Cofilin protein levels and distribution in dendritic spines in the SR. While overall protein and p-Cofilin levels were not altered in synaptosomal fractions (**Figures 5A,B**), immunogold staining showed a redistribution of p-Cofilin in Reelin-OE mice, passing from synaptic to extrasynaptic/intracellular locations when compared to controls (**Figures 5C–F**). The distribution of p-Cofilin in Reelin-OE-DOX mice was similar to that in controls (**Figure 5F**). These observations indicate that p-Cofilin redistributes in Reelin-OE mice, similar to NR2a/b receptor subunits.

### Gene Expression of Synaptic-Associated Genes Is Not Altered in Reelin-OE Mice

**FIGURE 6:**
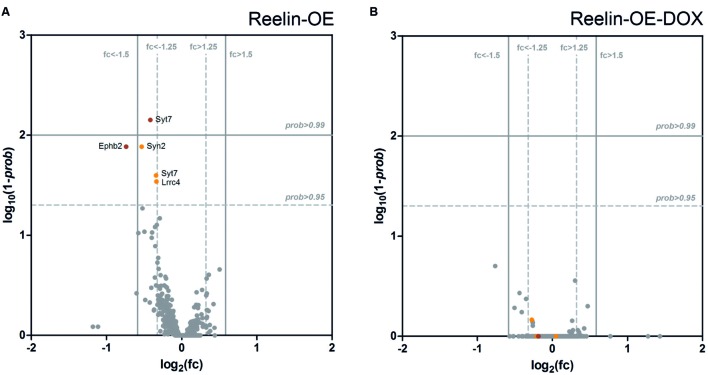
**Analysis of differential expression of genes directly associated with “Synapse” (GO:0045202) in the hippocampus of Reelin-OE mice. (A,B)** Volcano plots showing the magnitude of differential gene expression compared with control mice. Each dot represents an independent probe for a GO:0045202 gene. The vertical and horizontal lines mark the thresholds for defining up-/down-regulation for probability (*prob*) and fold change (fc), respectively. **(A)** Reelin-OE mice show most of the probes with non-differential expression (gray dots). Five probes show down-regulation (orange/red dots). **(B)** Reelin-OE mice treated for 1 week with doxycycline (Reelin-OE-DOX) do not show differences in gene expression with control mice.

The above protein expression experiments indicated that Reelin does not alter the expression of several important pre- and postsynaptic proteins in the adult brain. To confirm and extend these findings, we performed a genome-wide transcriptomic analysis, comparing mRNA abundance in the hippocampus of control, Reelin-OE, and Reelin-OE-DOX mice. In addition to the genes tested in Western blots, we screened for possible differential expression of 360 genes directly associated with the GO term “Synapse” (GO:0045202), which include presynaptic proteins and SNAREs, receptors, scaffolding and signaling proteins, and other synapse-related genes. As shown in **Figure 6** and **Supplementary Tables S1** and **S2**, the patterns of gene expression of these genes remained largely unaltered in Reelin-OE mice, compared to controls or Reelin-OE-DOX mice. Only a few gene probes (corresponding to Syt7, EphB2, Lrrc4, and Syn2 genes) showed minor, though statistically significant, regulation by Reelin levels. We conclude that the overall transcript expression for synapse-related genes is not altered by Reelin expression levels in the adult hippocampus.

## Discussion

### Reelin Confers Unique Structural Properties to Adult Hippocampal Dendritic Spines

Despite the role of Reelin in the developing brain in spine formation and maturation has been tackled consistently (Niu et al., 2004; Jossin and Goffinet, 2007; Niu et al., 2008), only recently it has started to be elucidated its impact on adult dendritic spine structure and physiology. In a previous study, we reported that spines of individual CA1 pyramidal cell dendrites in the SR of adult Reelin-OE mice were hypertrophied (Pujadas et al., 2010). These results were supported by studies in mDab1 KO mice showing that the size of dendritic spines was reduced (Trotter et al., 2013). Using a singular mouse model with increased Reelin expression specifically in the adult forebrain, here we show that sustained *in vivo* Reelin overexpression triggers larger dendritic spines and a lower circularity index (as an index of complexity) in all hippocampal layers, including the molecular layer of the DG. These observations thus support that these effects are a general consequence of the Reelin cascade in adult neurons. Enlargement of dendritic spines is a characteristic feature of synaptic plasticity mechanisms, including LTP (Toni et al., 2001; Popov et al., 2004; Yang et al., 2008; Hill and Zito, 2013), and is believed to confer a greater postsynaptic strength (Matsuzaki et al., 2004; Bourne and Harris, 2008; Lee et al., 2009; O’Donnell et al., 2011). In fact, Reelin-OE mice exhibit increased LTP responses *in vivo* (Pujadas et al., 2010). Moreover, synaptic plasticity is associated with changes in the proportion of dendritic spine types (Medvedev et al., 2010a,b). We found that Reelin overexpression in mice increases the percentage of dendritic spine types to type III (more than a 100% increase)—which most likely represent mushroom-type of dendritic spine—again in all layers (see Results). In contrast to thin dendritic spines, mushroom dendritic spines are considered to be the most mature spines, acting as stable “memory” spines (Matsuzaki et al., 2004; Bourne and Harris, 2007; Yasumatsu et al., 2008; Kasai et al., 2010). Moreover, recent studies report that LTP increases the proportion of mushroom spines (Medvedev et al., 2010a,b). Finally, we found a substantial increase in the percentage of spines bearing SAs and in the size of the SAs themselves in Reelin-OE mice. The SA is a Ca^2+^-sequestering organelle that regulates spine Ca^2+^ signaling, synaptic efficacy, and post-translational modification of receptors, among other processes, and that is enriched in mushroom spines (Spacek and Harris, 1997; Fiala et al., 2002; Nimchinsky et al., 2002; Korkotian et al., 2014). This finding again reinforces the notion that Reelin promotes the formation and stabilization of physiologically more efficient dendritic spines.

Reelin increases postsynaptic glutamatergic responses and LTP (Weeber et al., 2002; Beffert et al., 2005; Qiu et al., 2006a,b; Groc et al., 2007; Pujadas et al., 2010; Rogers et al., 2011, 2013). Moreover, infusion of Reelin enhances cognitive performance in wild-type mice (Rogers et al., 2011), while overexpression fully rescues cognitive deficits in mouse models of Alzheimer’s disease and during normal aging (Pujadas et al., 2014). Together with these findings, the present data suggest that one of the mechanisms by which Reelin enhances cognitive performance in a range of conditions, as well as potentiating glutamatergic neurotransmission, is by forming more stable and complex, mushroom-type dendritic spines, thus enabling stronger postsynaptic responses. Finally, the finding that dendritic spine phenotypes were dramatically reversed when the Reelin transgene was switched off supports the notion that the structural changes observed were effectively caused by the activation of the Reelin cascade.

### Reelin Exerts Presynaptic Effects in the Adult Brain

Reelin-deficient reeler mice exhibit decreased SNAP25 SNARE protein levels and reduced neurotransmitter release, which are rescued upon addition of recombinant Reelin (Hellwig et al., 2011). Here we found that Reelin overexpression did not alter the expression levels of SNAP25 protein or other SNARE components in Reelin-overexpressing adult mice. This observation suggests that while the absence of Reelin modifies SNAP25 levels, overactivation of the Reelin pathway is not sufficient to trigger increases in SNARE proteins. In contrast, here we report that axon terminals in the hippocampus of Reelin-OE mice were larger and exhibited increased complexity. Taken together with previous findings showing a higher density of axon terminals in reelin-OE mice (Pujadas et al., 2010), our results suggest that presynaptic numbers and complexity could be regulated independently from gene expression and protein synthesis. Possible mechanisms may include re-distribution of synaptic proteins and activation of synaptic proteins by post-translational modifications (such as phosphorylation) that may led to presynaptic functional and structural changes. In fact, Reelin increases short-term synaptic facilitation, a process believed to be caused essentially by presynaptic mechanisms (Pujadas et al., 2010; Rogers et al., 2011). Finally, a novel Reelin-dependent presynaptic mechanism has recently been reported in which the Reelin pathway stimulates spontaneous, action potential-independent neurotransmission via a Ca^2+^/VAMP7-dependent signaling cascade (Bal et al., 2013).

### Reelin Levels Do Not Regulate Synaptic Protein Expression Levels but do Determine Their Synaptic/Extrasynaptic Distribution

Here we show that Reelin levels do not regulate the expression of genes encoding for synaptic proteins, including presynaptic proteins such as SNAREs, glutamatergic receptor subunits, and postsynaptic scaffolding, cytoskeletal, and signaling proteins. Of a collection of 396 genes analyzed, only 4 genes (Syt7, Ephb2, Lrrc4, and Syn2) showed a moderate increase (~1.2-fold) in expression. These results were validated at the protein levels for several SNAREs, glutamate receptor subunits, and postsynaptic signaling proteins. Although the Reelin pathway is believed to activate the CREB transcription factor pathway (Rogers et al., 2011) and gene expression alterations have been described in reeler mice (Kuvbachieva et al., 2004) the present study supports that Reelin does not control the expression of target genes coding for synaptic proteins, at least in adult mice.

Recent studies show that Reelin increases synaptic activity through altering NMDA and AMPA receptor activation and surface trafficking activity *in vitro* (Beffert et al., 2005; Chen et al., 2005; Qiu et al., 2006b; Groc et al., 2007; Qiu and Weeber, 2007; Campo et al., 2009). Our results in the adult hippocampus *in vivo* support these observations by showing that Reelin induces a decrease in NR2a/b postsynaptic subunits, concomitant with an increase in extrasynaptic and cytosolic NR2a/b content. The fact that no significant differences are found in the total number of immunogold dots in spines for NR2a or NR2b proteins (data not shown) or in receptor subunit expression by WBs, suggests that Reelin leads to a re-distribution of the existing proteins rather than to *de novo* protein synthesis. Again, this Reelin-dependent localization was reversed after blocking Reelin transgene expression for 1 week, thereby suggesting that the Reelin pathway promotes both the lateral diffusion and internalization of these proteins *in vivo*. Notably, this recovery was found much more robust for NR2B than NR2A subunits. Taking into account that Reelin has been shown to trigger the mobility of NR2B subunits (Groc et al., 2007) and that Reelin overexpression *in vivo* recovers the chronic stress-induced reduction in hippocampal NR2B-mediated currents (Teixeira et al., 2011), our results support the notion that the Reelin pathway might interact differently with both NMDA receptor subunits. The fine mechanisms by which Reelin regulates NMDAR trafficking are still poorly understood, although they may include receptor subunit phosphorylation and the modulation of scaffolding and signaling proteins. It has been proposed that Reelin activates LIMK1, which phosphorylates Cofilin at serine residues (Meng et al., 2004; Chai et al., 2009), which in turn stabilizes the actin cytoskeleton in dendritic spines (Shi et al., 2009) and increases AMPAR surface trafficking (Gu et al., 2010; Wang et al., 2013). Although we did not detect differences in LIMK1 and Cofilin phosphorylation levels in Reelin-OE mice, we did observe Reelin-dependent changes in p-Cofilin distribution that mimicked those of NMDA receptor subunits. Thus, taken together with the above findings, the present findings in adult Reelin-OE mice support that, rather than inducing changes in the expression of synaptic protein components, Reelin signaling modulates glutamatergic neurotransmission by regulating post-translational mechanisms, including the trafficking and assembly of receptor subunits, Ca^2+^-dependent neurotransmitter release, and structural modifications of pre- and postsynaptic spine components.

### Is There a Layer-Specific Synaptic Responsiveness to Reelin?

Most of the pre- and postsynaptic alterations reported here in Reelin transgenic mice [including pre- and postsynaptic areas, length of contacts, area of SA and percentage of spines containing SA, and those observed in our previous study (Pujadas et al., 2010)] were more dramatic in the SLM, OML, and MML layers. These three layers correspond to principal cell dendrites receiving entorhinal input, in contrast to the SR, SO, and inner molecular layer (IML), which receive commissural/associational input. These differences could be explained by a differential, layer-specific distribution of Reelin signaling components or of Reelin itself. However, neither transgenic Reelin in our mice (Pujadas et al., 2010) nor the APOER2/VLDL receptors or mDab1 show clear layer-specific distributions (Borrell et al., 2007; Trotter et al., 2013). However, we cannot exclude that other signaling components are enriched in particular dendritic domains of pyramidal and granule cell neurons.

Perhaps the most dramatic layer-specific synaptic alteration reported here refers to the Reelin-dependent distribution of Synaptopodin. An actin-binding protein tightly associated with SA, Synaptopodin is critical for the formation of this organelle, which has been linked to the structural and physiological properties of dendritic spines (Deller et al., 2000, 2003, 2006; Zhang et al., 2013). Here we found that Reelin induces a dramatic shift in Synaptopodin distribution, with it being enriched in the SLM while decreasing in the dentate molecular layer. These alterations in distribution were not accompanied by changes in gene or protein expression. Although the present experiments do not allow us to offer a mechanistic explanation for these findings, taken together with the above observations, our results support the notion that Reelin modulates synaptic structure and function differentially in distinct dendritic domains of the same neuron and that this modulation is correlated with the type of afferent synaptic input.

## Conclusion

Our results highlight the participation of Reelin in the structural modulation of synaptic terminals and dendritic spines *in vivo* in adult mice. Importantly, they support the idea that, in addition to modulating presynaptic terminals, Reelin promotes the appearance of large, mushroom-type dendritic spines with large SAs and increased extrasynaptic NMDA receptors. Altogether, these data provide the structural basis to unravel the contribution of Reelin to normal neurotransmission and synaptic facilitation/LTP forms of plasticity and also to pathological conditions.

## Author Contributions

Conceived and designed the experiments: CB, ES, and AMa. Performed the experiments: CB, LP, AMu, and AMa. Analyzed the data: CB, ES, AMu, and AMa. Wrote the paper: CB, ES, and AMa.

## Conflict of Interest Statement

The authors declare that the research was conducted in the absence of any commercial or financial relationships that could be construed as a potential conflict of interest.

## Supplementary Material

FIGURE S1 **Histograms illustrating the percentage of dendritic spines types I and II in all the hippocampal layers in control, Reelin-OE, and DOX-treated Reelin-OE mice (mean ± SEM; **p* < 0.05, ***p* < 0.01, ****p* < 0.001 for control vs. Reelin-OE mice; squares for Reelin-OE vs. Reelin-OE+DOX; triangles for control vs. Reelin-OE+DOX mice; ANOVA test)**. IML, inner molecular layer; MML, medial molecular layer, OML, outer molecular layer; SLM, stratum lacunosum-moleculare; SO, stratum oriens; SP, stratum pyramida

FIGURE S2 **Western blot analysis and quantification of Synaptopodin. (A)** Immunoblot analysis of Synaptopodin in Reelin-OE, DOX-treated Reelin-OE mice and control littermates. **(B)** Histogram showing densitometric analysis (*n* = 3 animals per group) by fold change in the three groups of mice (mean ± SEM; ANOVA test).

FIGURE S3 **Western blot analysis and quantification of distinct post-synaptic proteins. (A–G)** Immunoblot analysis and its corresponding quantification of NR1 **(A)**, GluR1 **(B)**, GluR2/3 **(C)**, PSD-95 **(D)**, CaMKII **(E)**, LIMK1 **(F)**, and p-LIMK1/LIMK2 **(G)** from synaptosomal fractions of the hippocampus (*n* = 3) in control, Reelin-OE, and DOX-treated Reelin-OE mice. Values in histograms are normalized to the levels of actin and expressed as percentage of control mice (mean ± SEM; ANOVA test).

TABLE S1 **Gene expression of GO synapse genes**. The fold-change (fc) and the probability (prob) of differential expression are presented for all the probes in the Genechip Mouse Genome 430 2.0 array corresponding to GO:0045202 genes. Reelin-OE mice (R-OE) and Reelin-OE mice treated for one week with doxycycline (R-OE+1w(DOX)) were compared with controls.

TABLE S2 **Gene expression of selected genes**. The fold-change (fc) and the probability (prob) of differential expression are presented for all the probes in the Genechip Mouse Genome 430 2.0 array corresponding to Limk1/Limk2/Cfl1/Clf2 genes. Reelin-OE mice (R-OE) and Reelin-OE mice treated for one week with doxycycline (R-OE+1w(DOX)) were compared with controls.
