# Supplementary figures and images for: Reelin Exerts Structural, Biochemical and Transcriptional Regulation Over Presynaptic and Postsynaptic Elements in the Adult Hippocampus
Source: Front Cell Neurosci. 2016 May 30;10:138. doi: 10.3389/fncel.2016.00138 (PMC4884741; doi:10.3389/fncel.2016.00138)

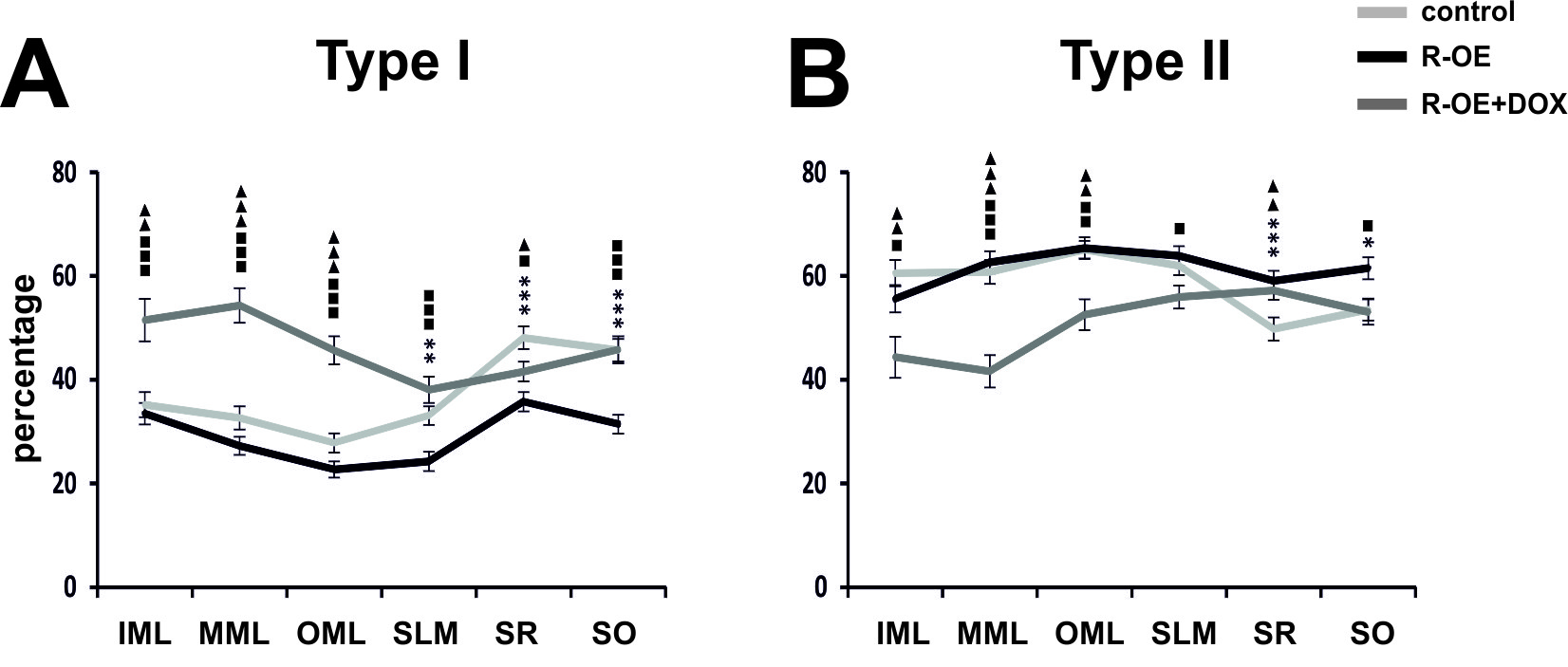

Supplement: FIGURE S1 — Histograms illustrating the percentage of dendritic spines types I and II in all the hippocampal layers in control, Reelin-OE, and DOX-treated Reelin-OE mice (mean ± SEM; *p < 0.05, **p < 0.01, ***p < 0.001 for control vs. Reelin-OE mice; squares for Reelin-OE vs. Reelin-OE+DOX; triangles for control vs. Reelin-OE+DOX mice; ANOVA test). IML, inner molecular layer; MML, medial molecular layer, OML, outer molecular layer; SLM, stratum lacunosum-moleculare; SO, stratum oriens; SP, stratum pyramida [file Image_1.JPEG]

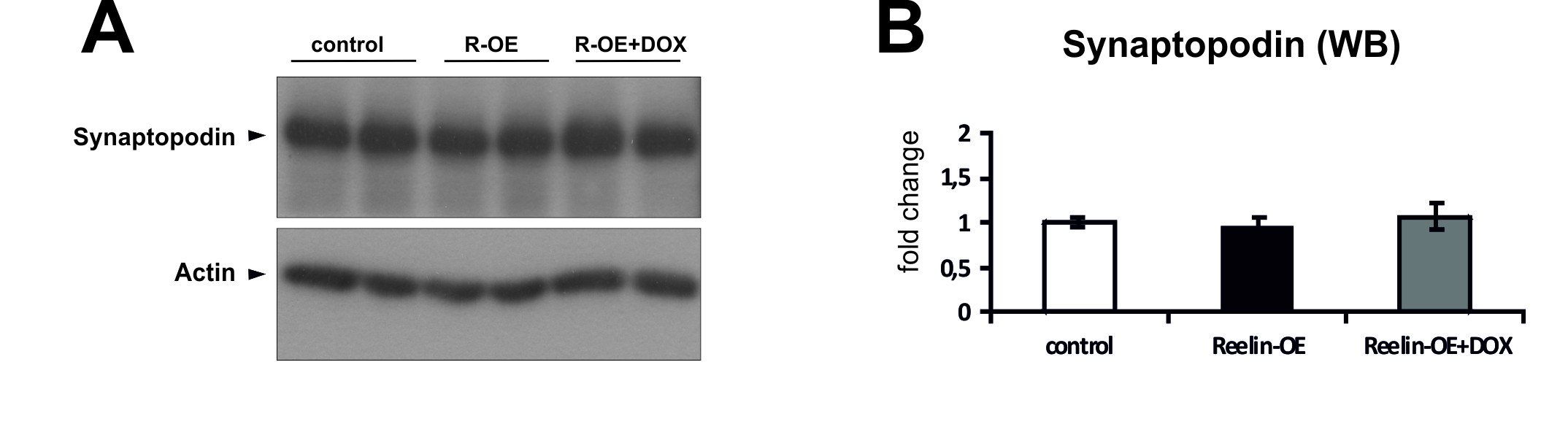

Supplement: FIGURE S2 — Western blot analysis and quantification of Synaptopodin. (A) Immunoblot analysis of Synaptopodin in Reelin-OE, DOX-treated Reelin-OE mice and control littermates. (B) Histogram showing densitometric analysis (n = 3 animals per group) by fold change in the three groups of mice (mean ± SEM; ANOVA test). [file Image_2.JPEG]

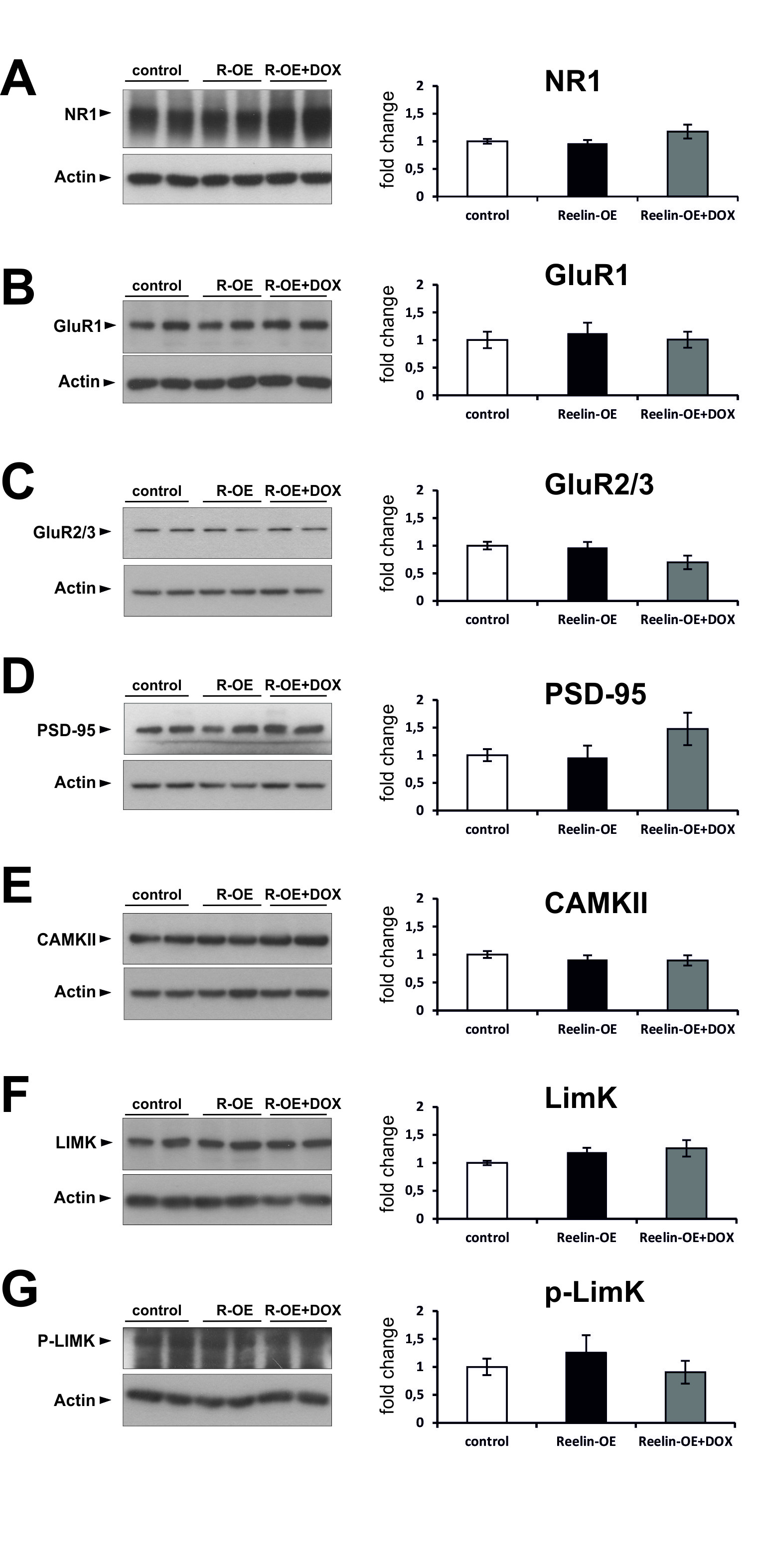

Supplement: FIGURE S3 — Western blot analysis and quantification of distinct post-synaptic proteins. (A–G) Immunoblot analysis and its corresponding quantification of NR1 (A), GluR1 (B), GluR2/3 (C), PSD-95 (D), CaMKII (E), LIMK1 (F), and p-LIMK1/LIMK2 (G) from synaptosomal fractions of the hippocampus (n = 3) in control, Reelin-OE, and DOX-treated Reelin-OE mice. Values in histograms are normalized to the levels of actin and expressed as percentage of control mice (mean ± SEM; ANOVA test). [file Image_3.JPEG]
