# Supplementary material for: Reelin Exerts Structural, Biochemical and Transcriptional Regulation Over Presynaptic and Postsynaptic Elements in the Adult Hippocampus
Source: Front Cell Neurosci. 2016 May 30;10:138. doi: 10.3389/fncel.2016.00138 (PMC4884741; doi:10.3389/fncel.2016.00138)
Supplement: TABLE S1 — Gene expression of GO synapse genes. The fold-change (fc) and the probability (prob) of differential expression are presented for all the probes in the Genechip Mouse Genome 430 2.0 array corresponding to GO:0045202 genes. Reelin-OE mice (R-OE) and Reelin-OE mice treated for one week with doxycycline (R-OE+1w(DOX)) were compared with controls. [file Table_1.DOCX]

| **Symbol** | **Gene name** | **Accession** | **Control - Reelin-OE** | | **Control - Reelin-OE-DOX** | |
| --- | --- | --- | --- | --- | --- | --- |
|  |  |  | **Fold change** | **P** | **Fold change** | **P** |
| Abi1 | abl-interactor 1 | 1423177_a_at | -1,002 | 0,000 | -1,023 | 0,000 |
| Abi1 | abl-interactor 1 | 1423178_at | -1,056 | 0,089 | -1,134 | 0,000 |
| Abi1 | abl-interactor 1 | 1438506_s_at | 1,069 | 0,000 | 1,070 | 0,000 |
| Abi1 | abl-interactor 1 | 1450890_a_at | 1,016 | 0,000 | 1,061 | 0,000 |
| Ache | acetylcholinesterase | 1422635_at | -1,027 | 0,000 | 1,108 | 0,000 |
| Add1 | adducin 1 (alpha) | 1420953_at | 1,078 | 0,086 | 1,049 | 0,000 |
| Add1 | adducin 1 (alpha) | 1420954_a_at | -1,065 | 0,084 | 1,110 | 0,000 |
| Add1 | adducin 1 (alpha) | 1450054_at | -1,014 | 0,000 | 1,006 | 0,000 |
| Adrbk1 | adrenergic receptor kinase, beta 1 | 1426249_at | 1,074 | 0,074 | 1,067 | 0,000 |
| Adrbk1 | adrenergic receptor kinase, beta 1 | 1451992_at | 1,072 | 0,016 | 1,050 | 0,000 |
| Adrbk2 | adrenergic receptor kinase, beta 2 | 1434450_s_at | 1,035 | 0,000 | -1,006 | 0,000 |
| Adrbk2 | adrenergic receptor kinase, beta 2 | 1439178_at | -1,030 | 0,000 | -1,071 | 0,000 |
| Adrbk2 | adrenergic receptor kinase, beta 2 | 1440801_s_at | 1,007 | 0,000 | -1,086 | 0,000 |
| Agrn | agrin | 1426670_at | -1,055 | 0,273 | 1,015 | 0,000 |
| Agrn | agrin | 1443980_at | -1,146 | 0,333 | -1,097 | 0,000 |
| Amph | amphiphysin | 1427044_a_at | -1,006 | 0,000 | 1,028 | 0,000 |
| Ank2 | ankyrin 2, brain | 1434264_at | 1,022 | 0,000 | 1,049 | 0,000 |
| Ank2 | ankyrin 2, brain | 1434265_s_at | 1,019 | 0,000 | 1,016 | 0,000 |
| Ank2 | ankyrin 2, brain | 1440042_at | 1,182 | 0,255 | 1,228 | 0,000 |
| Ank2 | ankyrin 2, brain | 1440043_at | -1,039 | 0,000 | 1,044 | 0,000 |
| Ank2 | ankyrin 2, brain | 1444023_at | 1,254 | 0,168 | 1,334 | 0,000 |
| Ank2 | ankyrin 2, brain | 1459317_at | 1,018 | 0,000 | 1,109 | 0,000 |
| Ank3 | ankyrin 3, epithelial | 1425202_a_at | -1,030 | 0,000 | 1,044 | 0,000 |
| Ank3 | ankyrin 3, epithelial | 1447259_at | -1,101 | 0,186 | 1,108 | 0,000 |
| Ank3 | ankyrin 3, epithelial | 1451628_a_at | -1,089 | 0,337 | -1,003 | 0,000 |
| Ank3 | ankyrin 3, epithelial | 1452124_at | 1,029 | 0,000 | 1,070 | 0,000 |
| Ank3 | ankyrin 3, epithelial | 1452872_at | 1,013 | 0,000 | 1,050 | 0,000 |
| Ank3 | ankyrin 3, epithelial | 1457288_at | -1,106 | 0,174 | 1,106 | 0,000 |
| Anks1b | ankyrin repeat and sterile alpha motif domain containing 1B | 1447464_at | -1,126 | 0,258 | -1,053 | 0,000 |
| Anks1b | ankyrin repeat and sterile alpha motif domain containing 1B | 1449634_a_at | 1,046 | 0,000 | 1,086 | 0,000 |
| Anks1b | ankyrin repeat and sterile alpha motif domain containing 1B | 1452938_at | -1,050 | 0,028 | -1,017 | 0,000 |
| Anks1b | ankyrin repeat and sterile alpha motif domain containing 1B | 1457060_at | -1,164 | 0,305 | 1,008 | 0,000 |
| Anks1b | ankyrin repeat and sterile alpha motif domain containing 1B | 1457990_at | -1,047 | 0,091 | -1,024 | 0,000 |
| Anks1b | ankyrin repeat and sterile alpha motif domain containing 1B | 1460449_at | 1,013 | 0,000 | -1,024 | 0,000 |
| Apbb1 | amyloid beta (A4) precursor protein-binding, family B, member 1 | 1423892_at | 1,036 | 0,000 | 1,122 | 0,000 |
| Apbb1 | amyloid beta (A4) precursor protein-binding, family B, member 1 | 1423893_x_at | 1,071 | 0,000 | 1,077 | 0,000 |
| Apc | adenomatosis polyposis coli | 1420956_at | 1,003 | 0,000 | 1,004 | 0,000 |
| Apc | adenomatosis polyposis coli | 1420957_at | 1,050 | 0,000 | 1,230 | 0,000 |
| Apc | adenomatosis polyposis coli | 1435543_at | -1,036 | 0,058 | 1,040 | 0,000 |
| Apc | adenomatosis polyposis coli | 1450056_at | 1,031 | 0,000 | 1,202 | 0,000 |
| Arc | activity regulated cytoskeletal-associated protein | 1418687_at | -1,515 | 0,621 | -1,309 | 0,000 |
| Arfgap1 | ADP-ribosylation factor GTPase activating protein 1 | 1427245_at | -1,140 | 0,394 | -1,060 | 0,000 |
| Arfgef2 | ADP-ribosylation factor guanine nucleotide-exchange factor 2 (brefeldin A-inhibited) | 1436192_at | -1,093 | 0,228 | -1,089 | 0,000 |
| Arfgef2 | ADP-ribosylation factor guanine nucleotide-exchange factor 2 (brefeldin A-inhibited) | 1443842_at | -1,131 | 0,187 | -1,204 | 0,000 |
| Arfgef2 | ADP-ribosylation factor guanine nucleotide-exchange factor 2 (brefeldin A-inhibited) | 1454982_at | 1,029 | 0,000 | 1,000 | 0,000 |
| Arhgap32 | Rho GTPase activating protein 32 | 1438451_at | 1,000 | 0,000 | 1,146 | 0,000 |
| Arhgap32 | Rho GTPase activating protein 32 | 1453709_at | -1,138 | 0,258 | -1,098 | 0,000 |
| Arr3 | arrestin 3, retinal | 1425232_x_at | -1,075 | 0,030 | -1,316 | 0,000 |
| Arr3 | arrestin 3, retinal | 1441144_at | -1,136 | 0,028 | -1,484 | 0,000 |
| Arr3 | arrestin 3, retinal | 1450329_a_at | -1,186 | 0,194 | -1,366 | 0,000 |
| Atad1 | ATPase family, AAA domain containing 1 | 1417555_at | -1,105 | 0,235 | -1,052 | 0,000 |
| Atad1 | ATPase family, AAA domain containing 1 | 1446838_at | 1,005 | 0,000 | -1,009 | 0,000 |
| Atad1 | ATPase family, AAA domain containing 1 | 1448763_at | 1,042 | 0,000 | 1,027 | 0,000 |
| Atad1 | ATPase family, AAA domain containing 1 | 1456382_at | 1,106 | 0,000 | 1,036 | 0,000 |
| Atcay | ataxia, cerebellar, Cayman type homolog (human) | 1434466_at | 1,050 | 0,000 | 1,048 | 0,000 |
| Atcay | ataxia, cerebellar, Cayman type homolog (human) | 1434467_at | 1,063 | 0,002 | 1,167 | 0,000 |
| Atcay | ataxia, cerebellar, Cayman type homolog (human) | 1454972_at | -1,022 | 0,000 | -1,025 | 0,000 |
| Atp1a2 | ATPase, Na+/K+ transporting, alpha 2 polypeptide | 1427465_at | -1,052 | 0,024 | -1,067 | 0,000 |
| Atp1a2 | ATPase, Na+/K+ transporting, alpha 2 polypeptide | 1434893_at | 1,012 | 0,000 | -1,071 | 0,000 |
| Atp1a2 | ATPase, Na+/K+ transporting, alpha 2 polypeptide | 1443823_s_at | 1,001 | 0,000 | -1,027 | 0,000 |
| Atp1a2 | ATPase, Na+/K+ transporting, alpha 2 polypeptide | 1452308_a_at | 1,080 | 0,000 | 1,043 | 0,000 |
| Atp1a2 | ATPase, Na+/K+ transporting, alpha 2 polypeptide | 1455136_at | 1,046 | 0,000 | -1,043 | 0,000 |
| Atp1a3 | ATPase, Na+/K+ transporting, alpha 3 polypeptide | 1424856_at | 1,061 | 0,000 | 1,026 | 0,000 |
| Atp1a3 | ATPase, Na+/K+ transporting, alpha 3 polypeptide | 1427481_a_at | -1,080 | 0,295 | 1,001 | 0,000 |
| Bcan | brevican | 1416718_at | -1,020 | 0,000 | -1,022 | 0,000 |
| Bcan | brevican | 1441899_x_at | -1,062 | 0,033 | -1,008 | 0,000 |
| Bcl2l1 | BCL2-like 1 | 1420887_a_at | 1,061 | 0,000 | -1,061 | 0,000 |
| Bcl2l1 | BCL2-like 1 | 1420888_at | -1,004 | 0,000 | -1,119 | 0,000 |
| Bcl2l1 | BCL2-like 1 | 1426050_at | 1,019 | 0,000 | 1,015 | 0,000 |
| Bcl2l1 | BCL2-like 1 | 1426191_a_at | 1,062 | 0,000 | -1,027 | 0,000 |
| Brsk1 | BR serine/threonine kinase 1 | 1439892_at | 1,068 | 0,000 | 1,091 | 0,000 |
| Bsn | bassoon | 1422023_at | -1,134 | 0,445 | 1,001 | 0,000 |
| Bsn | bassoon | 1436123_at | -1,035 | 0,052 | 1,029 | 0,000 |
| Bsn | bassoon | 1450467_at | -1,019 | 0,000 | 1,036 | 0,000 |
| Cacnb4 | calcium channel, voltage-dependent, beta 4 subunit | 1428928_at | -1,002 | 0,000 | 1,066 | 0,000 |
| Cacnb4 | calcium channel, voltage-dependent, beta 4 subunit | 1436912_at | -1,033 | 0,000 | 1,074 | 0,000 |
| Cacnb4 | calcium channel, voltage-dependent, beta 4 subunit | 1452089_at | 1,032 | 0,000 | 1,108 | 0,000 |
| Cacng5 | calcium channel, voltage-dependent, gamma subunit 5 | 1426330_at | -1,107 | 0,223 | -1,111 | 0,000 |
| Cacng5 | calcium channel, voltage-dependent, gamma subunit 5 | 1434785_at | -1,101 | 0,049 | -1,273 | 0,000 |
| Cacng8 | calcium channel, voltage-dependent, gamma subunit 8 | 1451864_at | -1,093 | 0,170 | -1,053 | 0,000 |
| Cacng8 | calcium channel, voltage-dependent, gamma subunit 8 | 1459579_at | -1,041 | 0,023 | -1,113 | 0,000 |
| Cadm1 | cell adhesion molecule 1 | 1417376_a_at | -1,002 | 0,000 | -1,096 | 0,000 |
| Cadm1 | cell adhesion molecule 1 | 1417377_at | -1,049 | 0,058 | -1,063 | 0,000 |
| Cadm1 | cell adhesion molecule 1 | 1417378_at | 1,011 | 0,000 | -1,020 | 0,000 |
| Cadm1 | cell adhesion molecule 1 | 1431611_a_at | -1,087 | 0,148 | -1,087 | 0,000 |
| Cadm2 | cell adhesion molecule 2 | 1429211_at | 1,104 | 0,153 | 1,171 | 0,000 |
| Cadm2 | cell adhesion molecule 2 | 1435145_at | 1,072 | 0,000 | 1,170 | 0,000 |
| Cadm2 | cell adhesion molecule 2 | 1435146_s_at | 1,032 | 0,000 | 1,146 | 0,000 |
| Cadm2 | cell adhesion molecule 2 | 1435147_x_at | -1,099 | 0,403 | -1,057 | 0,000 |
| Cadm2 | cell adhesion molecule 2 | 1436743_at | 1,090 | 0,000 | 1,146 | 0,000 |
| Cadm2 | cell adhesion molecule 2 | 1439048_at | -1,009 | 0,000 | 1,131 | 0,000 |
| Cadm2 | cell adhesion molecule 2 | 1459761_x_at | -1,032 | 0,041 | 1,011 | 0,000 |
| Cadps | Ca2+-dependent secretion activator | 1448955_s_at | 1,031 | 0,000 | 1,003 | 0,000 |
| Cadps2 | Ca2+-dependent activator protein for secretion 2 | 1451499_at | 1,021 | 0,000 | -1,017 | 0,000 |
| Calb1 | calbindin 1 | 1417504_at | 1,029 | 0,000 | -1,077 | 0,000 |
| Calb1 | calbindin 1 | 1448738_at | 1,026 | 0,000 | 1,046 | 0,000 |
| Calb1 | calbindin 1 | 1456934_at | -1,011 | 0,000 | -1,117 | 0,000 |
| Calb1 | calbindin 1 | 1458836_at | -1,245 | 0,602 | -1,243 | 0,000 |
| Camk2a | calcium/calmodulin-dependent protein kinase II alpha | 1437125_at | -1,126 | 0,211 | 1,054 | 0,000 |
| Camk2a | calcium/calmodulin-dependent protein kinase II alpha | 1441734_at | -1,268 | 0,683 | -1,262 | 0,000 |
| Camk2a | calcium/calmodulin-dependent protein kinase II alpha | 1442707_at | -1,059 | 0,276 | 1,088 | 0,000 |
| Camk2a | calcium/calmodulin-dependent protein kinase II alpha | 1452453_a_at | -1,044 | 0,000 | 1,232 | 0,000 |
| Camk2a | calcium/calmodulin-dependent protein kinase II alpha | 1457311_at | -1,054 | 0,198 | -1,011 | 0,000 |
| Camk2n1 | calcium/calmodulin-dependent protein kinase II inhibitor 1 | 1440455_at | -1,435 | 0,946 | -1,418 | 0,480 |
| Camk2n1 | calcium/calmodulin-dependent protein kinase II inhibitor 1 | 1456609_at | -1,036 | 0,060 | -1,010 | 0,000 |
| Cask | calcium/calmodulin-dependent serine protein kinase (MAGUK family) | 1422518_at | 1,041 | 0,000 | 1,008 | 0,000 |
| Cask | calcium/calmodulin-dependent serine protein kinase (MAGUK family) | 1422519_at | 1,134 | 0,244 | 1,161 | 0,000 |
| Cask | calcium/calmodulin-dependent serine protein kinase (MAGUK family) | 1427692_a_at | -1,103 | 0,281 | 1,033 | 0,000 |
| Cask | calcium/calmodulin-dependent serine protein kinase (MAGUK family) | 1455406_at | -1,009 | 0,000 | 1,046 | 0,000 |
| Cbln1 | cerebellin 1 precursor protein | 1423286_at | 1,126 | 0,000 | 1,212 | 0,000 |
| Cbln1 | cerebellin 1 precursor protein | 1423287_at | 1,055 | 0,000 | 1,216 | 0,000 |
| Cbln1 | cerebellin 1 precursor protein | 1423288_s_at | 1,089 | 0,000 | 1,308 | 0,000 |
| Cbln3 | cerebellin 3 precursor protein | 1422911_at | 1,035 | 0,000 | 1,047 | 0,000 |
| Cbln3 | cerebellin 3 precursor protein | 1455445_at | -1,021 | 0,000 | -1,045 | 0,000 |
| Cbln4 | cerebellin 4 precursor protein | 1433607_at | -1,115 | 0,091 | -1,032 | 0,000 |
| Cdh2 | cadherin 2 | 1418815_at | -1,020 | 0,000 | -1,069 | 0,000 |
| Cdh2 | cadherin 2 | 1449244_at | -1,002 | 0,000 | 1,061 | 0,000 |
| Cdh23 | cadherin 23 (otocadherin) | 1432346_a_at | 1,103 | 0,013 | 1,095 | 0,000 |
| Cdh23 | cadherin 23 (otocadherin) | 1452028_a_at | -1,057 | 0,073 | -1,100 | 0,000 |
| Cdk16 | cyclin-dependent kinase 16 | 1415956_a_at | 1,008 | 0,000 | 1,058 | 0,000 |
| Cdk16 | cyclin-dependent kinase 16 | 1438314_at | -1,103 | 0,091 | 1,102 | 0,000 |
| Cdk16 | cyclin-dependent kinase 16 | 1438625_s_at | -1,007 | 0,000 | 1,017 | 0,000 |
| Cdk16 | cyclin-dependent kinase 16 | 1443296_at | 1,034 | 0,000 | -1,164 | 0,000 |
| Cdk16 | cyclin-dependent kinase 16 | 1460169_a_at | 1,011 | 0,000 | -1,027 | 0,000 |
| Cdk5 | cyclin-dependent kinase 5 | 1422590_at | -1,082 | 0,140 | -1,021 | 0,000 |
| Cdk5 | cyclin-dependent kinase 5 | 1450674_at | 1,040 | 0,000 | 1,013 | 0,000 |
| Chn2 | chimerin (chimaerin) 2 | 1428573_at | -1,006 | 0,000 | 1,100 | 0,000 |
| Chn2 | chimerin (chimaerin) 2 | 1428574_a_at | 1,155 | 0,130 | 1,138 | 0,000 |
| Chrm1 | cholinergic receptor, muscarinic 1, CNS | 1439611_at | -1,010 | 0,000 | 1,057 | 0,000 |
| Chrm1 | cholinergic receptor, muscarinic 1, CNS | 1450833_at | 1,151 | 0,367 | 1,167 | 0,000 |
| Chrm3 | cholinergic receptor, muscarinic 3, cardiac | 1422258_at | -1,156 | 0,352 | 1,156 | 0,000 |
| Chrm4 | cholinergic receptor, muscarinic 4 | 1450575_at | -1,013 | 0,000 | -1,015 | 0,000 |
| Chrna1 | cholinergic receptor, nicotinic, alpha polypeptide 1 (muscle) | 1418852_at | -1,047 | 0,000 | -1,030 | 0,000 |
| Chrna2 | cholinergic receptor, nicotinic, alpha polypeptide 2 (neuronal) | 1425409_at | 1,015 | 0,000 | -1,086 | 0,000 |
| Chrna3 | cholinergic receptor, nicotinic, alpha polypeptide 3 | 1444368_at | -1,182 | 0,435 | -1,084 | 0,000 |
| Chrna3 | cholinergic receptor, nicotinic, alpha polypeptide 3 | 1452010_at | -1,129 | 0,095 | 1,135 | 0,000 |
| Chrna3 | cholinergic receptor, nicotinic, alpha polypeptide 3 | 1455931_at | 1,188 | 0,000 | 1,116 | 0,000 |
| Chrna4 | cholinergic receptor, nicotinic, alpha polypeptide 4 | 1421202_at | -1,110 | 0,101 | -1,036 | 0,000 |
| Chrna4 | cholinergic receptor, nicotinic, alpha polypeptide 4 | 1421203_at | 1,032 | 0,000 | 1,027 | 0,000 |
| Chrna4 | cholinergic receptor, nicotinic, alpha polypeptide 4 | 1456354_at | -1,076 | 0,076 | -1,066 | 0,000 |
| Chrna5 | cholinergic receptor, nicotinic, alpha polypeptide 5 | 1427401_at | 1,121 | 0,155 | 1,281 | 0,091 |
| Chrna5 | cholinergic receptor, nicotinic, alpha polypeptide 5 | 1442035_at | -1,043 | 0,000 | 1,149 | 0,000 |
| Chrna6 | cholinergic receptor, nicotinic, alpha polypeptide 6 | 1450426_at | -1,077 | 0,092 | -1,019 | 0,000 |
| Chrna6 | cholinergic receptor, nicotinic, alpha polypeptide 6 | 1450427_at | -1,063 | 0,073 | -1,026 | 0,000 |
| Chrna7 | cholinergic receptor, nicotinic, alpha polypeptide 7 | 1440681_at | -1,088 | 0,382 | -1,037 | 0,000 |
| Chrna7 | cholinergic receptor, nicotinic, alpha polypeptide 7 | 1450299_at | -1,274 | 0,872 | -1,009 | 0,000 |
| Chrna9 | cholinergic receptor, nicotinic, alpha polypeptide 9 | 1430086_at | -1,056 | 0,076 | -1,094 | 0,000 |
| Chrna9 | cholinergic receptor, nicotinic, alpha polypeptide 9 | 1445615_at | -1,116 | 0,119 | -1,009 | 0,000 |
| Chrnb1 | cholinergic receptor, nicotinic, beta polypeptide 1 (muscle) | 1420682_at | 1,107 | 0,160 | -1,083 | 0,000 |
| Chrnb2 | cholinergic receptor, nicotinic, beta polypeptide 2 (neuronal) | 1420744_at | -1,067 | 0,054 | -1,078 | 0,000 |
| Chrnb2 | cholinergic receptor, nicotinic, beta polypeptide 2 (neuronal) | 1436428_at | -1,150 | 0,213 | -1,058 | 0,000 |
| Chrnb2 | cholinergic receptor, nicotinic, beta polypeptide 2 (neuronal) | 1441837_at | -1,108 | 0,101 | 1,088 | 0,000 |
| Chrnb3 | cholinergic receptor, nicotinic, beta polypeptide 3 | 1451842_a_at | -1,061 | 0,087 | 1,129 | 0,000 |
| Chrnb4 | cholinergic receptor, nicotinic, beta polypeptide 4 | 1425849_at | 1,066 | 0,000 | 1,342 | 0,000 |
| Chrnb4 | cholinergic receptor, nicotinic, beta polypeptide 4 | 1457008_at | 1,358 | 0,000 | 1,710 | 0,000 |
| Chrnd | cholinergic receptor, nicotinic, delta polypeptide | 1420761_at | -1,127 | 0,251 | 1,013 | 0,000 |
| Chrne | cholinergic receptor, nicotinic, epsilon polypeptide | 1420560_at | -1,005 | 0,000 | 1,056 | 0,000 |
| Chrng | cholinergic receptor, nicotinic, gamma polypeptide | 1427728_at | 1,023 | 0,000 | 1,146 | 0,000 |
| Chrng | cholinergic receptor, nicotinic, gamma polypeptide | 1449532_at | -1,064 | 0,085 | -1,119 | 0,000 |
| Chrng | cholinergic receptor, nicotinic, gamma polypeptide | 1452520_a_at | -1,220 | 0,468 | -1,130 | 0,000 |
| Clstn1 | calsyntenin 1 | 1421860_at | 1,054 | 0,000 | 1,037 | 0,000 |
| Clstn1 | calsyntenin 1 | 1421861_at | 1,002 | 0,000 | 1,008 | 0,000 |
| Cnih3 | cornichon homolog 3 (Drosophila) | 1419517_at | -1,162 | 0,740 | -1,199 | 0,217 |
| Cntn2 | contactin 2 | 1435165_at | 1,117 | 0,281 | 1,154 | 0,000 |
| Cntn2 | contactin 2 | 1435166_at | -1,011 | 0,000 | 1,201 | 0,302 |
| Cntn2 | contactin 2 | 1450523_at | -1,006 | 0,000 | 1,008 | 0,000 |
| Cntn2 | contactin 2 | 1456962_at | 1,216 | 0,180 | -1,004 | 0,000 |
| Colq | collagen-like tail subunit (single strand of homotrimer) of asymmetric acetylcholinesterase | 1448081_at | -1,120 | 0,161 | -1,083 | 0,000 |
| Cpeb1 | cytoplasmic polyadenylation element binding protein 1 | 1417960_at | 1,023 | 0,000 | 1,156 | 0,000 |
| Cpeb3 | cytoplasmic polyadenylation element binding protein 3 | 1437765_at | 1,003 | 0,000 | 1,070 | 0,000 |
| Cpeb3 | cytoplasmic polyadenylation element binding protein 3 | 1445868_at | -1,278 | 0,917 | -1,084 | 0,000 |
| Cpeb3 | cytoplasmic polyadenylation element binding protein 3 | 1455372_at | -1,051 | 0,088 | -1,164 | 0,000 |
| Cpeb3 | cytoplasmic polyadenylation element binding protein 3 | 1456048_at | 1,052 | 0,000 | 1,111 | 0,000 |
| Cpeb4 | cytoplasmic polyadenylation element binding protein 4 | 1420617_at | 1,015 | 0,000 | -1,114 | 0,000 |
| Cpeb4 | cytoplasmic polyadenylation element binding protein 4 | 1420618_at | -1,045 | 0,069 | -1,190 | 0,000 |
| Cpeb4 | cytoplasmic polyadenylation element binding protein 4 | 1449931_at | -1,093 | 0,072 | -1,140 | 0,000 |
| Cplx1 | complexin 1 | 1417746_at | -1,036 | 0,078 | -1,000 | 0,000 |
| Cplx1 | complexin 1 | 1417747_at | 1,058 | 0,075 | 1,094 | 0,000 |
| Cplx1 | complexin 1 | 1448832_a_at | -1,073 | 0,173 | -1,017 | 0,000 |
| Cplx2 | complexin 2 | 1421477_at | -1,123 | 0,320 | -1,108 | 0,000 |
| Cplx2 | complexin 2 | 1436383_at | -1,036 | 0,002 | -1,052 | 0,000 |
| Cplx2 | complexin 2 | 1455672_s_at | -1,045 | 0,113 | -1,021 | 0,000 |
| Cplx3 | complexin 3 | 1424606_at | 1,174 | 0,000 | 1,263 | 0,000 |
| Cplx4 | complexin 4 | 1451590_at | 1,019 | 0,000 | 1,154 | 0,000 |
| Cpt1c | carnitine palmitoyltransferase 1c | 1435281_at | -1,068 | 0,145 | -1,055 | 0,000 |
| Cript | cysteine-rich PDZ-binding protein | 1423486_at | -1,008 | 0,000 | -1,016 | 0,000 |
| Cript | cysteine-rich PDZ-binding protein | 1423487_at | -1,229 | 0,393 | -1,310 | 0,000 |
| Ctbp2 | C-terminal binding protein 2 | 1422887_a_at | -1,005 | 0,000 | -1,018 | 0,000 |
| Ctbp2 | C-terminal binding protein 2 | 1434705_at | 1,034 | 0,000 | -1,090 | 0,000 |
| Ctnnb1 | catenin (cadherin associated protein), beta 1 | 1420811_a_at | 1,009 | 0,000 | -1,010 | 0,000 |
| Ctnnb1 | catenin (cadherin associated protein), beta 1 | 1430533_a_at | -1,098 | 0,276 | 1,161 | 0,000 |
| Ctnnb1 | catenin (cadherin associated protein), beta 1 | 1450008_a_at | -1,020 | 0,000 | 1,052 | 0,000 |
| Ctnnd1 | catenin (cadherin associated protein), delta 1 | 1422450_at | 1,059 | 0,000 | -1,079 | 0,000 |
| Ctnnd1 | catenin (cadherin associated protein), delta 1 | 1437448_s_at | 1,072 | 0,004 | -1,013 | 0,000 |
| Ctnnd1 | catenin (cadherin associated protein), delta 1 | 1445830_at | 1,082 | 0,022 | 1,135 | 0,000 |
| Cyfip1 | cytoplasmic FMR1 interacting protein 1 | 1416329_at | -1,083 | 0,414 | -1,040 | 0,000 |
| Cyfip1 | cytoplasmic FMR1 interacting protein 1 | 1457134_at | 1,061 | 0,000 | -1,056 | 0,000 |
| Cyfip1 | cytoplasmic FMR1 interacting protein 1 | 1459866_x_at | -1,146 | 0,593 | -1,068 | 0,000 |
| Cyfip2 | cytoplasmic FMR1 interacting protein 2 | 1428347_at | -1,017 | 0,000 | -1,076 | 0,000 |
| Cyfip2 | cytoplasmic FMR1 interacting protein 2 | 1442167_at | 1,058 | 0,000 | -1,099 | 0,000 |
| Cyfip2 | cytoplasmic FMR1 interacting protein 2 | 1449273_at | -1,064 | 0,158 | 1,051 | 0,000 |
| Dact1 | dapper homolog 1, antagonist of beta-catenin (xenopus) | 1417937_at | -1,006 | 0,000 | -1,275 | 0,577 |
| Dag1 | dystroglycan 1 | 1423872_a_at | -1,002 | 0,000 | 1,014 | 0,000 |
| Dag1 | dystroglycan 1 | 1426778_at | -1,022 | 0,000 | 1,000 | 0,000 |
| Dag1 | dystroglycan 1 | 1426779_x_at | 1,009 | 0,000 | -1,031 | 0,000 |
| Dag1 | dystroglycan 1 | 1456131_x_at | 1,049 | 0,000 | 1,071 | 0,000 |
| Dbh | dopamine beta hydroxylase | 1447592_at | -1,098 | 0,100 | -1,111 | 0,000 |
| Dbh | dopamine beta hydroxylase | 1450670_at | -1,160 | 0,217 | -1,132 | 0,000 |
| Dbh | dopamine beta hydroxylase | 1459848_x_at | -1,342 | 0,531 | -1,055 | 0,000 |
| Dbnl | drebrin-like | 1460334_at | 1,027 | 0,000 | 1,009 | 0,000 |
| Dennd1a | DENN/MADD domain containing 1A | 1424625_a_at | -1,085 | 0,229 | -1,011 | 0,000 |
| Dlg1 | discs, large homolog 1 (Drosophila) | 1445798_at | -1,173 | 0,282 | -1,042 | 0,000 |
| Dlg2 | discs, large homolog 2 (Drosophila) | 1421199_at | 1,119 | 0,000 | 1,253 | 0,000 |
| Dlg2 | discs, large homolog 2 (Drosophila) | 1421200_at | 1,121 | 0,007 | 1,263 | 0,000 |
| Dlg2 | discs, large homolog 2 (Drosophila) | 1437927_at | 1,022 | 0,000 | -1,004 | 0,000 |
| Dlg3 | discs, large homolog 3 (Drosophila) | 1416918_at | -1,010 | 0,000 | 1,029 | 0,000 |
| Dlg4 | discs, large homolog 4 (Drosophila) | 1419580_at | -1,045 | 0,069 | -1,026 | 0,000 |
| Dlg4 | discs, large homolog 4 (Drosophila) | 1419581_at | 1,062 | 0,000 | 1,191 | 0,000 |
| Dlg4 | discs, large homolog 4 (Drosophila) | 1460261_at | -1,133 | 0,360 | 1,038 | 0,000 |
| Dlgap1 | discs, large (Drosophila) homolog-associated protein 1 | 1429105_at | -1,033 | 0,000 | 1,041 | 0,000 |
| Dlgap1 | discs, large (Drosophila) homolog-associated protein 1 | 1436076_at | -1,073 | 0,174 | -1,050 | 0,000 |
| Dlgap1 | discs, large (Drosophila) homolog-associated protein 1 | 1438098_at | -1,160 | 0,493 | 1,081 | 0,000 |
| Dlgap1 | discs, large (Drosophila) homolog-associated protein 1 | 1440639_at | -1,055 | 0,076 | 1,068 | 0,000 |
| Dlgap1 | discs, large (Drosophila) homolog-associated protein 1 | 1443508_at | -1,081 | 0,062 | -1,029 | 0,000 |
| Dlgap1 | discs, large (Drosophila) homolog-associated protein 1 | 1453027_at | -1,034 | 0,054 | 1,008 | 0,000 |
| Dlgap2 | discs, large (Drosophila) homolog-associated protein 2 | 1439767_at | -1,017 | 0,000 | 1,104 | 0,000 |
| Dlgap3 | discs, large (Drosophila) homolog-associated protein 3 | 1436381_at | -1,097 | 0,169 | -1,031 | 0,000 |
| Dlgap4 | discs, large homolog-associated protein 4 (Drosophila) | 1426465_at | -1,116 | 0,422 | 1,050 | 0,000 |
| Dlgap4 | discs, large homolog-associated protein 4 (Drosophila) | 1455548_at | -1,025 | 0,000 | 1,062 | 0,000 |
| Dmd | dystrophin, muscular dystrophy | 1417307_at | -1,106 | 0,287 | -1,017 | 0,000 |
| Dmd | dystrophin, muscular dystrophy | 1430320_at | -1,082 | 0,091 | 1,086 | 0,000 |
| Dmd | dystrophin, muscular dystrophy | 1443315_at | -1,106 | 0,126 | 1,055 | 0,000 |
| Dmd | dystrophin, muscular dystrophy | 1448665_at | -1,085 | 0,217 | -1,007 | 0,000 |
| Dmxl2 | Dmx-like 2 | 1428749_at | 1,022 | 0,000 | 1,021 | 0,000 |
| Dnaja3 | DnaJ (Hsp40) homolog, subfamily A, member 3 | 1420629_a_at | -1,020 | 0,000 | 1,078 | 0,000 |
| Dnaja3 | DnaJ (Hsp40) homolog, subfamily A, member 3 | 1432066_at | -1,117 | 0,139 | -1,078 | 0,000 |
| Dnaja3 | DnaJ (Hsp40) homolog, subfamily A, member 3 | 1449935_a_at | 1,026 | 0,000 | -1,024 | 0,000 |
| Dnm1l | dynamin 1-like | 1428008_at | 1,078 | 0,000 | 1,086 | 0,000 |
| Dnm1l | dynamin 1-like | 1428086_at | -1,008 | 0,000 | 1,025 | 0,000 |
| Dnm1l | dynamin 1-like | 1428087_at | 1,000 | 0,000 | 1,076 | 0,000 |
| Dnm1l | dynamin 1-like | 1452638_s_at | -1,003 | 0,000 | 1,230 | 0,000 |
| Dnm2 | dynamin 2 | 1423629_at | 1,058 | 0,000 | 1,076 | 0,000 |
| Dnm2 | dynamin 2 | 1425135_a_at | -1,026 | 0,000 | 1,013 | 0,000 |
| Dnm2 | dynamin 2 | 1425136_x_at | 1,001 | 0,000 | 1,031 | 0,000 |
| Dnm2 | dynamin 2 | 1432004_a_at | -1,012 | 0,000 | 1,047 | 0,000 |
| Dnm2 | dynamin 2 | 1432005_at | 1,281 | 0,056 | 1,063 | 0,000 |
| Dnm2 | dynamin 2 | 1437938_x_at | -1,044 | 0,006 | 1,002 | 0,000 |
| Dnm2 | dynamin 2 | 1451057_x_at | -1,091 | 0,201 | -1,029 | 0,000 |
| Dnmbp | dynamin binding protein | 1427033_at | -1,141 | 0,371 | -1,144 | 0,000 |
| Dnmbp | dynamin binding protein | 1431244_s_at | -1,096 | 0,081 | -1,108 | 0,000 |
| Doc2a | double C2, alpha | 1436862_at | 1,081 | 0,023 | 1,046 | 0,000 |
| Dok7 | docking protein 7 | 1434812_s_at | -1,039 | 0,000 | -1,068 | 0,000 |
| Dok7 | docking protein 7 | 1442869_at | 1,021 | 0,000 | -1,018 | 0,000 |
| Dtna | dystrobrevin alpha | 1419223_a_at | -1,064 | 0,091 | 1,185 | 0,000 |
| Dtna | dystrobrevin alpha | 1425292_at | -1,002 | 0,000 | -1,022 | 0,000 |
| Dtna | dystrobrevin alpha | 1426066_a_at | -1,057 | 0,091 | 1,082 | 0,000 |
| Dtna | dystrobrevin alpha | 1427588_a_at | 1,015 | 0,000 | 1,031 | 0,000 |
| Dtna | dystrobrevin alpha | 1429768_at | -1,050 | 0,091 | 1,021 | 0,000 |
| Dtna | dystrobrevin alpha | 1453625_at | -1,007 | 0,000 | -1,016 | 0,000 |
| Dtna | dystrobrevin alpha | 1456069_at | 1,029 | 0,000 | 1,012 | 0,000 |
| Dtnb | dystrobrevin, beta | 1437651_a_at | 1,144 | 0,505 | 1,089 | 0,000 |
| Dtnb | dystrobrevin, beta | 1445329_at | 1,092 | 0,000 | 1,149 | 0,000 |
| Dtnbp1 | dystrobrevin binding protein 1 | 1431619_a_at | 1,163 | 0,466 | 1,144 | 0,000 |
| Dvl1 | dishevelled, dsh homolog 1 (Drosophila) | 1437301_a_at | -1,036 | 0,091 | -1,006 | 0,000 |
| Dvl1 | dishevelled, dsh homolog 1 (Drosophila) | 1450978_at | 1,015 | 0,000 | 1,000 | 0,000 |
| Efnb1 | ephrin B1 | 1418285_at | -1,020 | 0,000 | -1,171 | 0,000 |
| Efnb1 | ephrin B1 | 1418286_a_at | -1,020 | 0,000 | -1,022 | 0,000 |
| Efnb1 | ephrin B1 | 1451591_a_at | -1,073 | 0,083 | -1,077 | 0,000 |
| Egflam | EGF-like, fibronectin type III and laminin G domains | 1434647_at | -1,002 | 0,000 | 1,008 | 0,000 |
| Enah | enabled homolog (Drosophila) | 1421624_a_at | -1,050 | 0,034 | 1,118 | 0,000 |
| Enah | enabled homolog (Drosophila) | 1424800_at | -1,055 | 0,149 | -1,034 | 0,000 |
| Enah | enabled homolog (Drosophila) | 1424801_at | 1,014 | 0,000 | -1,009 | 0,000 |
| Enah | enabled homolog (Drosophila) | 1431162_a_at | -1,036 | 0,000 | 1,152 | 0,000 |
| Enah | enabled homolog (Drosophila) | 1442223_at | -1,016 | 0,000 | 1,010 | 0,000 |
| Epha4 | Eph receptor A4 | 1421928_at | -1,089 | 0,234 | 1,009 | 0,000 |
| Epha4 | Eph receptor A4 | 1421929_at | -1,234 | 0,675 | -1,019 | 0,000 |
| Epha4 | Eph receptor A4 | 1429021_at | -1,029 | 0,003 | -1,051 | 0,000 |
| Epha4 | Eph receptor A4 | 1439757_s_at | -1,216 | 0,592 | -1,024 | 0,000 |
| Epha4 | Eph receptor A4 | 1456863_at | -1,187 | 0,514 | 1,023 | 0,000 |
| Ephb2 | Eph receptor B2 | 1425015_at | -1,318 | 0,894 | -1,128 | 0,000 |
| Ephb2 | Eph receptor B2 | 1425016_at | -1,241 | 0,561 | -1,009 | 0,000 |
| Ephb2 | Eph receptor B2 | 1454022_at | -1,669 | 0,987 | -1,137 | 0,000 |
| Eps8 | epidermal growth factor receptor pathway substrate 8 | 1422824_s_at | 1,043 | 0,000 | -1,018 | 0,000 |
| Erc2 | ELKS/RAB6-interacting/CAST family member 2 | 1434582_at | -1,050 | 0,115 | -1,095 | 0,000 |
| Erc2 | ELKS/RAB6-interacting/CAST family member 2 | 1444681_at | -1,240 | 0,378 | -1,312 | 0,000 |
| Exoc4 | exocyst complex component 4 | 1422684_a_at | -1,030 | 0,012 | 1,001 | 0,000 |
| Exoc4 | exocyst complex component 4 | 1422685_at | 1,026 | 0,000 | 1,073 | 0,000 |
| Exoc4 | exocyst complex component 4 | 1422686_s_at | 1,018 | 0,000 | 1,057 | 0,000 |
| Exoc4 | exocyst complex component 4 | 1431062_a_at | -1,047 | 0,000 | 1,149 | 0,000 |
| Exoc4 | exocyst complex component 4 | 1453977_at | 1,058 | 0,000 | -1,238 | 0,000 |
| Faim2 | Fas apoptotic inhibitory molecule 2 | 1429518_at | 1,013 | 0,000 | 1,017 | 0,000 |
| Faim2 | Fas apoptotic inhibitory molecule 2 | 1455410_at | 1,060 | 0,000 | 1,021 | 0,000 |
| Farp1 | FERM, RhoGEF (Arhgef) and pleckstrin domain protein 1 (chondrocyte-derived) | 1452280_at | 1,051 | 0,000 | -1,065 | 0,000 |
| Farp1 | FERM, RhoGEF (Arhgef) and pleckstrin domain protein 1 (chondrocyte-derived) | 1459799_at | -1,042 | 0,000 | -1,064 | 0,000 |
| Fbxo45 | F-box protein 45 | 1428742_at | -1,053 | 0,091 | 1,055 | 0,000 |
| Fbxo45 | F-box protein 45 | 1452895_at | -1,057 | 0,251 | -1,018 | 0,000 |
| Fmr1 | fragile X mental retardation syndrome 1 homolog | 1423369_at | -1,015 | 0,000 | 1,021 | 0,000 |
| Fmr1 | fragile X mental retardation syndrome 1 homolog | 1426086_a_at | 1,013 | 0,000 | 1,278 | 0,000 |
| Fmr1 | fragile X mental retardation syndrome 1 homolog | 1452550_a_at | 1,044 | 0,000 | 1,067 | 0,000 |
| Gabbr1 | gamma-aminobutyric acid (GABA) B receptor, 1 | 1422051_a_at | -1,023 | 0,000 | 1,049 | 0,000 |
| Gabbr1 | gamma-aminobutyric acid (GABA) B receptor, 1 | 1425595_at | 1,081 | 0,120 | -1,018 | 0,000 |
| Gabbr1 | gamma-aminobutyric acid (GABA) B receptor, 1 | 1437188_at | -1,008 | 0,000 | -1,014 | 0,000 |
| Gabbr1 | gamma-aminobutyric acid (GABA) B receptor, 1 | 1455021_at | 1,028 | 0,000 | -1,032 | 0,000 |
| Gabra1 | gamma-aminobutyric acid (GABA) A receptor, subunit alpha 1 | 1421280_at | -1,017 | 0,000 | -1,034 | 0,000 |
| Gabra1 | gamma-aminobutyric acid (GABA) A receptor, subunit alpha 1 | 1421281_at | -1,130 | 0,303 | -1,092 | 0,000 |
| Gabra1 | gamma-aminobutyric acid (GABA) A receptor, subunit alpha 1 | 1436889_at | -1,015 | 0,000 | 1,008 | 0,000 |
| Gabra1 | gamma-aminobutyric acid (GABA) A receptor, subunit alpha 1 | 1455766_at | -1,122 | 0,507 | -1,105 | 0,000 |
| Gabra2 | gamma-aminobutyric acid (GABA) A receptor, subunit alpha 2 | 1420299_at | -1,009 | 0,000 | -1,029 | 0,000 |
| Gabra2 | gamma-aminobutyric acid (GABA) A receptor, subunit alpha 2 | 1421738_at | -1,038 | 0,000 | 1,298 | 0,000 |
| Gabra2 | gamma-aminobutyric acid (GABA) A receptor, subunit alpha 2 | 1443865_at | -1,033 | 0,000 | 1,342 | 0,164 |
| Gabra2 | gamma-aminobutyric acid (GABA) A receptor, subunit alpha 2 | 1449807_x_at | 1,004 | 0,000 | 1,068 | 0,000 |
| Gabra2 | gamma-aminobutyric acid (GABA) A receptor, subunit alpha 2 | 1455444_at | -1,055 | 0,090 | 1,075 | 0,000 |
| Gabra3 | gamma-aminobutyric acid (GABA) A receptor, subunit alpha 3 | 1421263_at | -1,054 | 0,000 | 1,075 | 0,000 |
| Gabra3 | gamma-aminobutyric acid (GABA) A receptor, subunit alpha 3 | 1436957_at | 1,072 | 0,000 | 1,059 | 0,000 |
| Gabra4 | gamma-aminobutyric acid (GABA) A receptor, subunit alpha 4 | 1429330_at | -1,116 | 0,343 | -1,037 | 0,000 |
| Gabra4 | gamma-aminobutyric acid (GABA) A receptor, subunit alpha 4 | 1433707_at | -1,068 | 0,130 | -1,096 | 0,000 |
| Gabra5 | gamma-aminobutyric acid (GABA) A receptor, subunit alpha 5 | 1433602_at | -1,103 | 0,416 | -1,165 | 0,000 |
| Gabra6 | gamma-aminobutyric acid (GABA) A receptor, subunit alpha 6 | 1417121_at | -1,171 | 0,026 | 1,052 | 0,000 |
| Gabra6 | gamma-aminobutyric acid (GABA) A receptor, subunit alpha 6 | 1451706_a_at | -1,012 | 0,000 | 1,089 | 0,000 |
| Gabrb1 | gamma-aminobutyric acid (GABA) A receptor, subunit beta 1 | 1419719_at | 1,004 | 0,000 | -1,032 | 0,000 |
| Gabrb2 | gamma-aminobutyric acid (GABA) A receptor, subunit beta 2 | 1428203_at | -1,129 | 0,642 | -1,038 | 0,000 |
| Gabrb2 | gamma-aminobutyric acid (GABA) A receptor, subunit beta 2 | 1428204_at | -1,147 | 0,473 | 1,002 | 0,000 |
| Gabrb2 | gamma-aminobutyric acid (GABA) A receptor, subunit beta 2 | 1428205_x_at | -1,099 | 0,282 | 1,030 | 0,000 |
| Gabrb2 | gamma-aminobutyric acid (GABA) A receptor, subunit beta 2 | 1429685_at | -1,120 | 0,213 | 1,059 | 0,000 |
| Gabrb2 | gamma-aminobutyric acid (GABA) A receptor, subunit beta 2 | 1450319_at | -1,002 | 0,000 | 1,091 | 0,000 |
| Gabrb3 | gamma-aminobutyric acid (GABA) A receptor, subunit beta 3 | 1421189_at | -1,090 | 0,092 | 1,021 | 0,000 |
| Gabrb3 | gamma-aminobutyric acid (GABA) A receptor, subunit beta 3 | 1421190_at | -1,223 | 0,403 | -1,310 | 0,000 |
| Gabrb3 | gamma-aminobutyric acid (GABA) A receptor, subunit beta 3 | 1435021_at | -1,020 | 0,000 | 1,073 | 0,000 |
| Gabrd | gamma-aminobutyric acid (GABA) A receptor, subunit delta | 1449980_a_at | -1,312 | 0,906 | -1,248 | 0,000 |
| Gabrd | gamma-aminobutyric acid (GABA) A receptor, subunit delta | 1457763_at | -1,140 | 0,279 | 1,046 | 0,000 |
| Gabre | gamma-aminobutyric acid (GABA) A receptor, subunit epsilon | 1421629_at | -1,107 | 0,172 | -1,103 | 0,000 |
| Gabrg1 | gamma-aminobutyric acid (GABA) A receptor, subunit gamma 1 | 1427227_at | 1,063 | 0,000 | -1,014 | 0,000 |
| Gabrg1 | gamma-aminobutyric acid (GABA) A receptor, subunit gamma 1 | 1460408_at | 1,028 | 0,000 | -1,107 | 0,000 |
| Gabrg2 | gamma-aminobutyric acid (GABA) A receptor, subunit gamma 2 | 1418177_at | -1,033 | 0,021 | 1,007 | 0,000 |
| Gabrg2 | gamma-aminobutyric acid (GABA) A receptor, subunit gamma 2 | 1437147_at | -1,096 | 0,243 | -1,062 | 0,000 |
| Gabrg3 | gamma-aminobutyric acid (GABA) A receptor, subunit gamma 3 | 1422187_at | -1,398 | 0,557 | -1,326 | 0,000 |
| Gabrg3 | gamma-aminobutyric acid (GABA) A receptor, subunit gamma 3 | 1439717_at | -1,211 | 0,521 | -1,271 | 0,000 |
| Gabrp | gamma-aminobutyric acid (GABA) A receptor, pi | 1424647_at | 1,075 | 0,000 | -1,048 | 0,000 |
| Gabrp | gamma-aminobutyric acid (GABA) A receptor, pi | 1451424_at | 1,014 | 0,000 | 1,036 | 0,000 |
| Gabrq | gamma-aminobutyric acid (GABA) A receptor, subunit theta | 1421536_at | 1,026 | 0,000 | 1,053 | 0,000 |
| Gabrr1 | gamma-aminobutyric acid (GABA) C receptor, subunit rho 1 | 1450300_at | -1,201 | 0,613 | -1,325 | 0,428 |
| Gabrr2 | gamma-aminobutyric acid (GABA) C receptor, subunit rho 2 | 1420735_at | 1,284 | 0,428 | -1,005 | 0,000 |
| Gad1 | glutamic acid decarboxylase 1 | 1416561_at | -1,019 | 0,000 | -1,051 | 0,000 |
| Gad1 | glutamic acid decarboxylase 1 | 1416562_at | -1,065 | 0,140 | -1,018 | 0,000 |
| Gad2 | glutamic acid decarboxylase 2 | 1421978_at | 1,247 | 0,203 | 1,131 | 0,000 |
| Gad2 | glutamic acid decarboxylase 2 | 1429589_at | -1,055 | 0,125 | 1,021 | 0,000 |
| Gap43 | growth associated protein 43 | 1423537_at | -1,095 | 0,195 | -1,086 | 0,000 |
| Glra1 | glycine receptor, alpha 1 subunit | 1422277_at | -1,096 | 0,131 | 1,065 | 0,000 |
| Glra1 | glycine receptor, alpha 1 subunit | 1437139_at | 1,027 | 0,000 | -1,006 | 0,000 |
| Glra2 | glycine receptor, alpha 2 subunit | 1434098_at | 1,255 | 0,601 | 1,049 | 0,000 |
| Glra3 | glycine receptor, alpha 3 subunit | 1450239_at | -1,079 | 0,059 | -1,146 | 0,000 |
| Glra4 | glycine receptor, alpha 4 subunit | 1451937_at | -1,009 | 0,000 | 1,108 | 0,000 |
| Glrb | glycine receptor, beta subunit | 1422504_at | 1,044 | 0,000 | 1,058 | 0,000 |
| Glrb | glycine receptor, beta subunit | 1459850_x_at | 1,060 | 0,000 | -1,045 | 0,000 |
| Gopc | golgi associated PDZ and coiled-coil motif containing | 1421191_s_at | 1,013 | 0,000 | -1,015 | 0,000 |
| Gopc | golgi associated PDZ and coiled-coil motif containing | 1450153_at | -1,038 | 0,000 | -1,034 | 0,000 |
| Gopc | golgi associated PDZ and coiled-coil motif containing | 1453221_at | -1,002 | 0,000 | 1,028 | 0,000 |
| Gphn | gephyrin | 1426462_at | -1,033 | 0,045 | 1,043 | 0,000 |
| Gphn | gephyrin | 1426463_at | 1,012 | 0,000 | 1,140 | 0,000 |
| Gphn | gephyrin | 1430038_at | -1,044 | 0,000 | -1,031 | 0,000 |
| Gphn | gephyrin | 1444498_at | -1,018 | 0,000 | -1,066 | 0,000 |
| Gria1 | glutamate receptor, ionotropic, AMPA1 (alpha 1) | 1435239_at | -1,009 | 0,000 | -1,045 | 0,000 |
| Gria1 | glutamate receptor, ionotropic, AMPA1 (alpha 1) | 1458285_at | -1,280 | 0,735 | -1,073 | 0,000 |
| Gria2 | glutamate receptor, ionotropic, AMPA2 (alpha 2) | 1421970_a_at | -1,031 | 0,000 | 1,021 | 0,000 |
| Gria2 | glutamate receptor, ionotropic, AMPA2 (alpha 2) | 1434146_at | -1,046 | 0,082 | -1,027 | 0,000 |
| Gria2 | glutamate receptor, ionotropic, AMPA2 (alpha 2) | 1453098_at | -1,036 | 0,000 | 1,030 | 0,000 |
| Gria3 | glutamate receptor, ionotropic, AMPA3 (alpha 3) | 1420563_at | -1,117 | 0,144 | -1,017 | 0,000 |
| Gria3 | glutamate receptor, ionotropic, AMPA3 (alpha 3) | 1434728_at | -1,132 | 0,595 | -1,091 | 0,000 |
| Gria4 | glutamate receptor, ionotropic, AMPA4 (alpha 4) | 1421351_at | 1,015 | 0,000 | 1,100 | 0,000 |
| Gria4 | glutamate receptor, ionotropic, AMPA4 (alpha 4) | 1435722_at | -1,125 | 0,300 | 1,016 | 0,000 |
| Gria4 | glutamate receptor, ionotropic, AMPA4 (alpha 4) | 1436772_at | -1,071 | 0,091 | -1,046 | 0,000 |
| Gria4 | glutamate receptor, ionotropic, AMPA4 (alpha 4) | 1440891_at | 1,051 | 0,000 | 1,064 | 0,000 |
| Grid1 | glutamate receptor, ionotropic, delta 1 | 1421569_at | -1,047 | 0,059 | -1,005 | 0,000 |
| Grid1 | glutamate receptor, ionotropic, delta 1 | 1441499_at | -1,010 | 0,000 | 1,008 | 0,000 |
| Grid2 | glutamate receptor, ionotropic, delta 2 | 1421435_at | -1,145 | 0,285 | -1,041 | 0,000 |
| Grid2 | glutamate receptor, ionotropic, delta 2 | 1421436_at | -1,025 | 0,000 | 1,084 | 0,000 |
| Grid2 | glutamate receptor, ionotropic, delta 2 | 1435487_at | 1,002 | 0,000 | 1,012 | 0,000 |
| Grid2 | glutamate receptor, ionotropic, delta 2 | 1437824_at | 1,003 | 0,000 | 1,017 | 0,000 |
| Grid2 | glutamate receptor, ionotropic, delta 2 | 1459245_s_at | 1,074 | 0,000 | 1,009 | 0,000 |
| Grid2ip | glutamate receptor, ionotropic, delta 2 (Grid2) interacting protein 1 | 1450310_at | -1,020 | 0,000 | 1,043 | 0,000 |
| Grik1 | glutamate receptor, ionotropic, kainate 1 | 1427676_a_at | -1,143 | 0,349 | -1,039 | 0,000 |
| Grik1 | glutamate receptor, ionotropic, kainate 1 | 1439987_at | -1,111 | 0,184 | 1,006 | 0,000 |
| Grik2 | glutamate receptor, ionotropic, kainate 2 (beta 2) | 1425790_a_at | -1,172 | 0,475 | -1,005 | 0,000 |
| Grik2 | glutamate receptor, ionotropic, kainate 2 (beta 2) | 1439286_at | -1,220 | 0,658 | -1,043 | 0,000 |
| Grik2 | glutamate receptor, ionotropic, kainate 2 (beta 2) | 1457683_at | -1,223 | 0,932 | -1,072 | 0,000 |
| Grik3 | glutamate receptor, ionotropic, kainate 3 | 1427709_at | 1,015 | 0,000 | 1,151 | 0,000 |
| Grik3 | glutamate receptor, ionotropic, kainate 3 | 1440177_at | -1,115 | 0,080 | -1,177 | 0,000 |
| Grik4 | glutamate receptor, ionotropic, kainate 4 | 1437681_at | -1,126 | 0,307 | 1,066 | 0,000 |
| Grik5 | glutamate receptor, ionotropic, kainate 5 (gamma 2) | 1418784_at | -1,028 | 0,000 | 1,077 | 0,000 |
| Grin1 | glutamate receptor, ionotropic, NMDA1 (zeta 1) | 1437968_at | -1,118 | 0,207 | -1,045 | 0,000 |
| Grin1 | glutamate receptor, ionotropic, NMDA1 (zeta 1) | 1450202_at | -1,033 | 0,000 | 1,048 | 0,000 |
| Grin2a | glutamate receptor, ionotropic, NMDA2A (epsilon 1) | 1421616_at | -1,186 | 0,302 | 1,040 | 0,000 |
| Grin2b | glutamate receptor, ionotropic, NMDA2B (epsilon 2) | 1422223_at | 1,054 | 0,000 | 1,272 | 0,000 |
| Grin2b | glutamate receptor, ionotropic, NMDA2B (epsilon 2) | 1431700_at | -1,036 | 0,000 | 1,110 | 0,000 |
| Grin2b | glutamate receptor, ionotropic, NMDA2B (epsilon 2) | 1457003_at | -1,090 | 0,319 | -1,010 | 0,000 |
| Grin2c | glutamate receptor, ionotropic, NMDA2C (epsilon 3) | 1449245_at | -1,060 | 0,021 | 1,023 | 0,000 |
| Grin2d | glutamate receptor, ionotropic, NMDA2D (epsilon 4) | 1421393_at | -1,007 | 0,000 | 1,038 | 0,000 |
| Grin2d | glutamate receptor, ionotropic, NMDA2D (epsilon 4) | 1442328_at | -1,099 | 0,093 | -1,012 | 0,000 |
| Grin3a | glutamate receptor ionotropic, NMDA3A | 1436575_at | 1,069 | 0,000 | -1,117 | 0,000 |
| Grin3a | glutamate receptor ionotropic, NMDA3A | 1438866_at | 1,109 | 0,133 | -1,209 | 0,000 |
| Grin3a | glutamate receptor ionotropic, NMDA3A | 1458378_at | 1,002 | 0,000 | -1,020 | 0,000 |
| Grin3b | glutamate receptor, ionotropic, NMDA3B | 1449899_at | -1,076 | 0,075 | -1,063 | 0,000 |
| Grip1 | glutamate receptor interacting protein 1 | 1421350_a_at | 1,075 | 0,000 | 1,165 | 0,000 |
| Grip1 | glutamate receptor interacting protein 1 | 1435951_at | 1,010 | 0,000 | 1,053 | 0,000 |
| Grm2 | glutamate receptor, metabotropic 2 | 1431344_at | -1,227 | 0,526 | -1,019 | 0,000 |
| Grm2 | glutamate receptor, metabotropic 2 | 1435607_at | -1,236 | 0,784 | -1,141 | 0,000 |
| Grm7 | glutamate receptor, metabotropic 7 | 1443119_at | -1,022 | 0,000 | -1,008 | 0,000 |
| Grm7 | glutamate receptor, metabotropic 7 | 1459532_at | -1,041 | 0,000 | 1,095 | 0,000 |
| Gsg1l | GSG1-like | 1436013_at | -1,410 | 0,908 | -1,180 | 0,000 |
| Hcn2 | hyperpolarization-activated, cyclic nucleotide-gated K+ 2 | 1421154_at | -1,201 | 0,381 | 1,116 | 0,000 |
| Hcrt | hypocretin | 1420471_at | -1,036 | 0,031 | 1,014 | 0,000 |
| Homer1 | homer homolog 1 (Drosophila) | 1421768_a_at | -1,076 | 0,032 | 1,168 | 0,000 |
| Homer1 | homer homolog 1 (Drosophila) | 1425671_at | 1,050 | 0,000 | 1,113 | 0,000 |
| Homer1 | homer homolog 1 (Drosophila) | 1425710_a_at | -1,080 | 0,023 | 1,230 | 0,000 |
| Homer1 | homer homolog 1 (Drosophila) | 1437363_at | -1,060 | 0,242 | 1,003 | 0,000 |
| Homer1 | homer homolog 1 (Drosophila) | 1439662_at | -1,079 | 0,357 | 1,027 | 0,000 |
| Homer2 | homer homolog 2 (Drosophila) | 1424367_a_at | 1,083 | 0,251 | 1,233 | 0,721 |
| Homer2 | homer homolog 2 (Drosophila) | 1436110_at | 1,029 | 0,000 | 1,302 | 0,016 |
| Homer2 | homer homolog 2 (Drosophila) | 1457671_at | 1,060 | 0,000 | 1,082 | 0,000 |
| Homer3 | homer homolog 3 (Drosophila) | 1424859_at | -1,054 | 0,066 | -1,067 | 0,000 |
| Htr2b | 5-hydroxytryptamine (serotonin) receptor 2B | 1422125_at | -1,137 | 0,373 | -1,021 | 0,000 |
| Htr3a | 5-hydroxytryptamine (serotonin) receptor 3A | 1418268_at | 1,120 | 0,156 | 1,089 | 0,000 |
| Ica1 | islet cell autoantigen 1 | 1417901_a_at | -1,009 | 0,000 | 1,068 | 0,000 |
| Ica1 | islet cell autoantigen 1 | 1431644_a_at | 1,053 | 0,000 | 1,093 | 0,000 |
| Igsf9 | immunoglobulin superfamily, member 9 | 1420518_a_at | -1,185 | 0,457 | -1,241 | 0,000 |
| Igsf9 | immunoglobulin superfamily, member 9 | 1441420_at | -1,030 | 0,000 | -1,002 | 0,000 |
| Insr | insulin receptor | 1421380_at | -1,015 | 0,000 | 1,004 | 0,000 |
| Insr | insulin receptor | 1434446_at | 1,083 | 0,103 | 1,032 | 0,000 |
| Insr | insulin receptor | 1450225_at | -1,237 | 0,530 | -1,316 | 0,000 |
| Itga3 | integrin alpha 3 | 1421997_s_at | 1,142 | 0,292 | 1,064 | 0,000 |
| Itga3 | integrin alpha 3 | 1455158_at | 1,039 | 0,000 | 1,001 | 0,000 |
| Itga3 | integrin alpha 3 | 1460305_at | 1,004 | 0,000 | -1,019 | 0,000 |
| Itga5 | integrin alpha 5 (fibronectin receptor alpha) | 1423267_s_at | 1,027 | 0,000 | 1,002 | 0,000 |
| Itga5 | integrin alpha 5 (fibronectin receptor alpha) | 1423268_at | 1,100 | 0,004 | 1,065 | 0,000 |
| Itga5 | integrin alpha 5 (fibronectin receptor alpha) | 1457561_at | 1,120 | 0,125 | 1,010 | 0,000 |
| Itga5 | integrin alpha 5 (fibronectin receptor alpha) | 1458996_at | 1,107 | 0,083 | 1,058 | 0,000 |
| Itgb1 | integrin beta 1 (fibronectin receptor beta) | 1426918_at | 1,148 | 0,628 | 1,002 | 0,000 |
| Itgb1 | integrin beta 1 (fibronectin receptor beta) | 1426919_at | -1,056 | 0,001 | -1,153 | 0,000 |
| Itgb1 | integrin beta 1 (fibronectin receptor beta) | 1426920_x_at | -1,022 | 0,000 | -1,034 | 0,000 |
| Itgb1 | integrin beta 1 (fibronectin receptor beta) | 1427771_x_at | 1,260 | 0,729 | 1,171 | 0,000 |
| Itgb1 | integrin beta 1 (fibronectin receptor beta) | 1438119_at | 1,029 | 0,000 | -1,054 | 0,000 |
| Itgb1 | integrin beta 1 (fibronectin receptor beta) | 1452545_a_at | 1,089 | 0,157 | 1,031 | 0,000 |
| Itsn1 | intersectin 1 (SH3 domain protein 1A) | 1421192_a_at | -1,032 | 0,000 | 1,077 | 0,000 |
| Itsn1 | intersectin 1 (SH3 domain protein 1A) | 1425899_a_at | 1,005 | 0,000 | 1,084 | 0,000 |
| Itsn1 | intersectin 1 (SH3 domain protein 1A) | 1435884_at | 1,106 | 0,156 | -1,018 | 0,000 |
| Itsn1 | intersectin 1 (SH3 domain protein 1A) | 1435885_s_at | 1,045 | 0,000 | -1,017 | 0,000 |
| Itsn1 | intersectin 1 (SH3 domain protein 1A) | 1436579_s_at | -1,076 | 0,198 | 1,023 | 0,000 |
| Itsn1 | intersectin 1 (SH3 domain protein 1A) | 1441034_at | -1,293 | 0,436 | -1,021 | 0,000 |
| Itsn1 | intersectin 1 (SH3 domain protein 1A) | 1442637_at | -1,073 | 0,029 | -1,042 | 0,000 |
| Itsn1 | intersectin 1 (SH3 domain protein 1A) | 1452338_s_at | -1,034 | 0,031 | -1,009 | 0,000 |
| Itsn1 | intersectin 1 (SH3 domain protein 1A) | 1459621_at | 1,014 | 0,000 | 1,011 | 0,000 |
| Kctd12 | potassium channel tetramerisation domain containing 12 | 1434881_s_at | -1,062 | 0,204 | -1,009 | 0,000 |
| Kctd16 | potassium channel tetramerisation domain containing 16 | 1429666_at | -1,117 | 0,225 | -1,075 | 0,000 |
| Kctd8 | potassium channel tetramerisation domain containing 8 | 1441495_at | -1,197 | 0,556 | -1,180 | 0,000 |
| Kctd8 | potassium channel tetramerisation domain containing 8 | 1455923_at | -1,197 | 0,390 | -1,177 | 0,000 |
| Klhl17 | kelch-like 17 (Drosophila) | 1435257_at | -1,096 | 0,143 | -1,039 | 0,000 |
| Lamb2 | laminin, beta 2 | 1416513_at | 1,125 | 0,023 | -1,035 | 0,000 |
| Lgi1 | leucine-rich repeat LGI family, member 1 | 1435851_at | -1,091 | 0,347 | -1,057 | 0,000 |
| Lgi3 | leucine-rich repeat LGI family, member 3 | 1433667_at | 1,008 | 0,000 | 1,146 | 0,000 |
| Lgi3 | leucine-rich repeat LGI family, member 3 | 1436238_at | 1,058 | 0,000 | 1,127 | 0,000 |
| Lgi3 | leucine-rich repeat LGI family, member 3 | 1460546_at | 1,111 | 0,278 | 1,211 | 0,133 |
| Lin7a | lin-7 homolog A (C. elegans) | 1435805_at | -1,059 | 0,165 | -1,005 | 0,000 |
| Lin7a | lin-7 homolog A (C. elegans) | 1438450_at | -1,136 | 0,351 | 1,129 | 0,000 |
| Lin7a | lin-7 homolog A (C. elegans) | 1456656_at | 1,035 | 0,000 | 1,022 | 0,000 |
| Lin7b | lin-7 homolog B (C. elegans) | 1418683_at | -1,113 | 0,436 | 1,030 | 0,000 |
| Lin7b | lin-7 homolog B (C. elegans) | 1439239_at | -1,098 | 0,389 | 1,008 | 0,000 |
| Lin7b | lin-7 homolog B (C. elegans) | 1439240_x_at | -1,134 | 0,565 | -1,056 | 0,000 |
| Lin7b | lin-7 homolog B (C. elegans) | 1449172_a_at | -1,172 | 0,684 | -1,060 | 0,000 |
| Lin7c | lin-7 homolog C (C. elegans) | 1418898_at | 1,083 | 0,000 | -1,050 | 0,000 |
| Lin7c | lin-7 homolog C (C. elegans) | 1423322_at | -1,035 | 0,000 | -1,053 | 0,000 |
| Lin7c | lin-7 homolog C (C. elegans) | 1449262_s_at | 1,131 | 0,000 | 1,052 | 0,000 |
| Lin7c | lin-7 homolog C (C. elegans) | 1450937_at | 1,028 | 0,000 | 1,006 | 0,000 |
| Lphn1 | latrophilin 1 | 1428510_at | -1,009 | 0,000 | -1,049 | 0,000 |
| Lphn1 | latrophilin 1 | 1452812_at | -1,025 | 0,000 | 1,056 | 0,000 |
| Lrfn1 | leucine rich repeat and fibronectin type III domain containing 1 | 1421364_at | -1,057 | 0,054 | 1,118 | 0,000 |
| Lrfn1 | leucine rich repeat and fibronectin type III domain containing 1 | 1444669_at | -1,150 | 0,538 | -1,138 | 0,000 |
| Lrfn2 | leucine rich repeat and fibronectin type III domain containing 2 | 1453126_at | -1,256 | 0,921 | -1,167 | 0,000 |
| Lrfn3 | leucine rich repeat and fibronectin type III domain containing 3 | 1456767_at | -1,034 | 0,000 | 1,022 | 0,000 |
| Lrp6 | low density lipoprotein receptor-related protein 6 | 1451022_at | -1,007 | 0,000 | 1,052 | 0,000 |
| Lrrc4 | leucine rich repeat containing 4 | 1416097_at | -1,263 | 0,971 | -1,155 | 0,000 |
| Lrrc4 | leucine rich repeat containing 4 | 1435832_at | -1,064 | 0,232 | -1,046 | 0,000 |
| Lrrc4c | leucine rich repeat containing 4C | 1437201_at | -1,146 | 0,666 | -1,063 | 0,000 |
| Lrrc4c | leucine rich repeat containing 4C | 1437825_at | -1,133 | 0,454 | -1,143 | 0,000 |
| Lrrc4c | leucine rich repeat containing 4C | 1456759_at | -1,065 | 0,057 | 1,011 | 0,000 |
| Lrrk2 | leucine-rich repeat kinase 2 | 1431394_a_at | 1,052 | 0,000 | 1,047 | 0,000 |
| Lrrtm1 | leucine rich repeat transmembrane neuronal 1 | 1437746_at | -1,123 | 0,349 | 1,028 | 0,000 |
| Lrrtm1 | leucine rich repeat transmembrane neuronal 1 | 1452624_at | -1,156 | 0,501 | -1,008 | 0,000 |
| Lrrtm1 | leucine rich repeat transmembrane neuronal 1 | 1455883_a_at | -1,145 | 0,590 | -1,043 | 0,000 |
| Lrrtm2 | leucine rich repeat transmembrane neuronal 2 | 1437787_at | 1,063 | 0,000 | 1,078 | 0,000 |
| Lrrtm2 | leucine rich repeat transmembrane neuronal 2 | 1455489_at | 1,166 | 0,502 | 1,091 | 0,000 |
| Lrrtm2 | leucine rich repeat transmembrane neuronal 2 | 1456637_at | 1,095 | 0,081 | 1,202 | 0,000 |
| Lrrtm3 | leucine rich repeat transmembrane neuronal 3 | 1434759_at | 1,013 | 0,000 | -1,158 | 0,000 |
| Lrrtm3 | leucine rich repeat transmembrane neuronal 3 | 1434760_at | -1,017 | 0,000 | -1,161 | 0,000 |
| Lrrtm3 | leucine rich repeat transmembrane neuronal 3 | 1434761_at | -1,088 | 0,093 | -1,138 | 0,000 |
| Lrrtm3 | leucine rich repeat transmembrane neuronal 3 | 1440112_at | -1,131 | 0,194 | -1,231 | 0,000 |
| Lrrtm4 | leucine rich repeat transmembrane neuronal 4 | 1437214_at | -1,490 | 0,905 | -1,695 | 0,801 |
| Lrrtm4 | leucine rich repeat transmembrane neuronal 4 | 1455937_at | -1,249 | 0,813 | -1,355 | 0,631 |
| Magi2 | membrane associated guanylate kinase, WW and PDZ domain containing 2 | 1420532_at | -1,209 | 0,680 | -1,033 | 0,000 |
| Magi2 | membrane associated guanylate kinase, WW and PDZ domain containing 2 | 1454855_at | -1,006 | 0,000 | -1,008 | 0,000 |
| Mapk8ip1 | mitogen-activated protein kinase 8 interacting protein 1 | 1425679_a_at | 1,084 | 0,064 | 1,179 | 0,000 |
| Mapk8ip1 | mitogen-activated protein kinase 8 interacting protein 1 | 1440619_at | 1,100 | 0,125 | 1,116 | 0,000 |
| Mdm2 | transformed mouse 3T3 cell double minute 2 | 1423605_a_at | 1,031 | 0,000 | -1,004 | 0,000 |
| Mdm2 | transformed mouse 3T3 cell double minute 2 | 1427718_a_at | 1,082 | 0,000 | 1,025 | 0,000 |
| Mdm2 | transformed mouse 3T3 cell double minute 2 | 1457929_at | -1,029 | 0,000 | 1,209 | 0,000 |
| Mff | mitochondrial fission factor | 1451572_a_at | 1,069 | 0,035 | -1,002 | 0,000 |
| Mff | mitochondrial fission factor | 1456582_x_at | -1,026 | 0,034 | -1,022 | 0,000 |
| Mff | mitochondrial fission factor | 1456736_x_at | -1,021 | 0,000 | -1,081 | 0,000 |
| Mgll | monoglyceride lipase | 1426785_s_at | -1,062 | 0,091 | 1,082 | 0,000 |
| Mgll | monoglyceride lipase | 1431331_at | 1,091 | 0,000 | 1,018 | 0,000 |
| Mgll | monoglyceride lipase | 1450391_a_at | -1,078 | 0,194 | 1,046 | 0,000 |
| Mgll | monoglyceride lipase | 1453836_a_at | 1,097 | 0,055 | 1,203 | 0,000 |
| Mink1 | misshapen-like kinase 1 (zebrafish) | 1449362_a_at | -1,035 | 0,024 | -1,004 | 0,000 |
| Mme | membrane metallo endopeptidase | 1422975_at | 1,105 | 0,000 | 1,191 | 0,000 |
| Mpdz | multiple PDZ domain protein | 1418663_at | -1,024 | 0,000 | 1,033 | 0,000 |
| Mpdz | multiple PDZ domain protein | 1418664_at | -1,087 | 0,181 | -1,021 | 0,000 |
| Mpdz | multiple PDZ domain protein | 1441361_at | 1,051 | 0,000 | -1,028 | 0,000 |
| Mpdz | multiple PDZ domain protein | 1442978_at | -1,168 | 0,226 | -1,097 | 0,000 |
| Mpst | mercaptopyruvate sulfurtransferase | 1418356_at | 1,261 | 0,618 | 1,232 | 0,000 |
| Musk | muscle, skeletal, receptor tyrosine kinase | 1450511_at | -1,070 | 0,098 | 1,193 | 0,000 |
| Myo6 | myosin VI | 1421120_at | 1,070 | 0,000 | 1,095 | 0,000 |
| Myo6 | myosin VI | 1433942_at | -1,003 | 0,000 | 1,060 | 0,000 |
| Myo6 | myosin VI | 1435559_at | -1,006 | 0,000 | 1,041 | 0,000 |
| Myo7a | myosin VIIA | 1421385_a_at | 1,295 | 0,215 | 1,124 | 0,000 |
| Myrip | myosin VIIA and Rab interacting protein | 1460601_at | -1,043 | 0,086 | -1,073 | 0,000 |
| Nbea | neurobeachin | 1452251_at | -1,007 | 0,000 | -1,023 | 0,000 |
| Ncs1 | neuronal calcium sensor 1 | 1434887_at | -1,027 | 0,043 | -1,038 | 0,000 |
| Ncs1 | neuronal calcium sensor 1 | 1450146_at | -1,039 | 0,000 | 1,036 | 0,000 |
| Ncs1 | neuronal calcium sensor 1 | 1460293_at | -1,072 | 0,095 | -1,074 | 0,000 |
| Neto1 | neuropilin (NRP) and tolloid (TLL)-like 1 | 1425132_at | -1,087 | 0,163 | 1,046 | 0,000 |
| Neto1 | neuropilin (NRP) and tolloid (TLL)-like 1 | 1456283_at | -1,104 | 0,386 | -1,039 | 0,000 |
| Nf2 | neurofibromatosis 2 | 1421820_a_at | -1,126 | 0,411 | -1,046 | 0,000 |
| Nf2 | neurofibromatosis 2 | 1427708_a_at | -1,146 | 0,563 | 1,109 | 0,000 |
| Nf2 | neurofibromatosis 2 | 1450382_at | -1,085 | 0,086 | 1,077 | 0,000 |
| Nf2 | neurofibromatosis 2 | 1451829_a_at | 1,048 | 0,000 | 1,105 | 0,000 |
| Nlgn1 | neuroligin 1 | 1421648_at | -1,074 | 0,043 | 1,061 | 0,000 |
| Nlgn1 | neuroligin 1 | 1437160_at | -1,035 | 0,014 | -1,005 | 0,000 |
| Nlgn2 | neuroligin 2 | 1455143_at | -1,016 | 0,000 | 1,038 | 0,000 |
| Nlgn2 | neuroligin 2 | 1460013_at | -1,040 | 0,000 | 1,060 | 0,000 |
| Nmnat2 | nicotinamide nucleotide adenylyltransferase 2 | 1436155_at | 1,052 | 0,046 | -1,007 | 0,000 |
| Nos1 | nitric oxide synthase 1, neuronal | 1422949_at | -1,090 | 0,161 | -1,171 | 0,000 |
| Nos1 | nitric oxide synthase 1, neuronal | 1429887_at | -1,119 | 0,261 | -1,148 | 0,000 |
| Nos1 | nitric oxide synthase 1, neuronal | 1438483_at | -1,046 | 0,029 | 1,016 | 0,000 |
| Nos1 | nitric oxide synthase 1, neuronal | 1458626_at | -1,103 | 0,185 | -1,014 | 0,000 |
| Nrcam | neuron-glia-CAM-related cell adhesion molecule | 1430583_at | -1,059 | 0,140 | 1,032 | 0,000 |
| Nrcam | neuron-glia-CAM-related cell adhesion molecule | 1434709_at | -1,042 | 0,091 | 1,025 | 0,000 |
| Nrcam | neuron-glia-CAM-related cell adhesion molecule | 1443281_at | 1,016 | 0,000 | 1,015 | 0,000 |
| Nrcam | neuron-glia-CAM-related cell adhesion molecule | 1458833_at | -1,169 | 0,183 | -1,018 | 0,000 |
| Nrg1 | neuregulin 1 | 1456524_at | 1,086 | 0,000 | -1,191 | 0,000 |
| Nrgn | neurogranin | 1423231_at | -1,087 | 0,242 | -1,017 | 0,000 |
| Nrn1 | neuritin 1 | 1428393_at | -1,030 | 0,045 | -1,042 | 0,000 |
| Nrxn1 | neurexin I | 1428240_at | -1,078 | 0,143 | 1,155 | 0,000 |
| Nrxn1 | neurexin I | 1439358_a_at | -1,050 | 0,091 | -1,051 | 0,000 |
| Nrxn1 | neurexin I | 1439359_x_at | -1,009 | 0,000 | -1,005 | 0,000 |
| Nrxn1 | neurexin I | 1442501_at | 1,135 | 0,000 | 1,085 | 0,000 |
| Nrxn1 | neurexin I | 1447216_at | -1,105 | 0,091 | 1,001 | 0,000 |
| Nrxn1 | neurexin I | 1454691_at | -1,020 | 0,000 | 1,122 | 0,000 |
| Olfm1 | olfactomedin 1 | 1425784_a_at | -1,094 | 0,559 | -1,020 | 0,000 |
| Olfm1 | olfactomedin 1 | 1426562_a_at | -1,079 | 0,431 | -1,019 | 0,000 |
| Olfm1 | olfactomedin 1 | 1455796_x_at | -1,055 | 0,091 | -1,025 | 0,000 |
| Olfm2 | olfactomedin 2 | 1435790_at | 1,419 | 0,780 | 1,303 | 0,000 |
| Olfm3 | olfactomedin 3 | 1425898_x_at | -1,006 | 0,000 | 1,149 | 0,000 |
| Olfm3 | olfactomedin 3 | 1426512_at | 1,067 | 0,000 | 1,073 | 0,000 |
| Olfm3 | olfactomedin 3 | 1452090_a_at | 1,015 | 0,000 | 1,122 | 0,000 |
| Ophn1 | oligophrenin 1 | 1419107_at | -1,081 | 0,256 | 1,137 | 0,000 |
| Ophn1 | oligophrenin 1 | 1419108_at | -1,078 | 0,149 | -1,030 | 0,000 |
| Ophn1 | oligophrenin 1 | 1456709_at | -1,160 | 0,084 | 1,069 | 0,000 |
| Otof | otoferlin | 1420419_a_at | 1,236 | 0,198 | -1,131 | 0,000 |
| P2rx4 | purinergic receptor P2X, ligand-gated ion channel 4 | 1425525_a_at | -1,022 | 0,000 | -1,003 | 0,000 |
| P2rx4 | purinergic receptor P2X, ligand-gated ion channel 4 | 1441772_at | -1,023 | 0,000 | -1,019 | 0,000 |
| P2rx4 | purinergic receptor P2X, ligand-gated ion channel 4 | 1446429_at | 1,119 | 0,000 | 1,026 | 0,000 |
| P2rx4 | purinergic receptor P2X, ligand-gated ion channel 4 | 1452527_a_at | 1,102 | 0,000 | 1,099 | 0,000 |
| P2rx7 | purinergic receptor P2X, ligand-gated ion channel, 7 | 1419853_a_at | 1,102 | 0,000 | 1,027 | 0,000 |
| P2rx7 | purinergic receptor P2X, ligand-gated ion channel, 7 | 1422218_at | -1,052 | 0,021 | -1,136 | 0,000 |
| P2rx7 | purinergic receptor P2X, ligand-gated ion channel, 7 | 1439787_at | 1,060 | 0,000 | 1,032 | 0,000 |
| Pacsin1 | protein kinase C and casein kinase substrate in neurons 1 | 1449380_at | -1,039 | 0,030 | -1,012 | 0,000 |
| Pacsin1 | protein kinase C and casein kinase substrate in neurons 1 | 1449381_a_at | 1,054 | 0,000 | 1,138 | 0,000 |
| Park2 | Parkinson disease (autosomal recessive, juvenile) 2, parkin | 1420755_a_at | 1,023 | 0,000 | 1,212 | 0,000 |
| Park2 | Parkinson disease (autosomal recessive, juvenile) 2, parkin | 1426135_a_at | -1,191 | 0,555 | 1,085 | 0,000 |
| Park2 | Parkinson disease (autosomal recessive, juvenile) 2, parkin | 1449975_a_at | -1,030 | 0,005 | -1,001 | 0,000 |
| Pcdh8 | protocadherin 8 | 1417051_at | -1,137 | 0,329 | -1,267 | 0,012 |
| Pcdh8 | protocadherin 8 | 1447825_x_at | -1,060 | 0,147 | -1,203 | 0,287 |
| Pclo | piccolo (presynaptic cytomatrix protein) | 1419392_at | 1,024 | 0,000 | 1,046 | 0,000 |
| Pclo | piccolo (presynaptic cytomatrix protein) | 1452423_at | -1,004 | 0,000 | 1,266 | 0,132 |
| Pclo | piccolo (presynaptic cytomatrix protein) | 1455373_at | 1,028 | 0,000 | 1,152 | 0,000 |
| Pdlim5 | PDZ and LIM domain 5 | 1421413_a_at | 1,036 | 0,000 | 1,159 | 0,000 |
| Pdlim5 | PDZ and LIM domain 5 | 1422861_s_at | 1,071 | 0,000 | -1,175 | 0,000 |
| Pdlim5 | PDZ and LIM domain 5 | 1422862_at | 1,131 | 0,000 | 1,051 | 0,000 |
| Pdlim5 | PDZ and LIM domain 5 | 1422863_s_at | 1,285 | 0,752 | 1,385 | 0,499 |
| Pdlim5 | PDZ and LIM domain 5 | 1427475_a_at | 1,088 | 0,179 | -1,053 | 0,000 |
| Pdlim5 | PDZ and LIM domain 5 | 1429783_at | -1,016 | 0,000 | -1,014 | 0,000 |
| Pdlim5 | PDZ and LIM domain 5 | 1450786_x_at | 1,087 | 0,032 | 1,065 | 0,000 |
| Pfn1 | profilin 1 | 1449018_at | 1,100 | 0,118 | 1,053 | 0,000 |
| Phactr1 | phosphatase and actin regulator 1 | 1432851_at | 1,061 | 0,000 | 1,270 | 0,000 |
| Phactr1 | phosphatase and actin regulator 1 | 1432852_at | 1,050 | 0,000 | 1,168 | 0,000 |
| Phactr1 | phosphatase and actin regulator 1 | 1439022_at | 1,019 | 0,000 | 1,068 | 0,000 |
| Phactr1 | phosphatase and actin regulator 1 | 1454832_at | 1,028 | 0,000 | 1,053 | 0,000 |
| Pi4k2a | phosphatidylinositol 4-kinase type 2 alpha | 1433462_a_at | -1,060 | 0,087 | 1,049 | 0,000 |
| Pias3 | protein inhibitor of activated STAT 3 | 1421646_a_at | 1,108 | 0,008 | 1,065 | 0,000 |
| Pias3 | protein inhibitor of activated STAT 3 | 1451115_at | 1,053 | 0,000 | 1,028 | 0,000 |
| Picalm | phosphatidylinositol binding clathrin assembly protein | 1446968_at | 1,202 | 0,384 | 1,108 | 0,000 |
| Picalm | phosphatidylinositol binding clathrin assembly protein | 1451316_a_at | 1,032 | 0,000 | -1,020 | 0,000 |
| Pick1 | protein interacting with C kinase 1 | 1419384_at | 1,104 | 0,274 | 1,062 | 0,000 |
| Pja2 | praja 2, RING-H2 motif containing | 1424442_a_at | 1,013 | 0,000 | 1,043 | 0,000 |
| Pja2 | praja 2, RING-H2 motif containing | 1427148_at | 1,046 | 0,000 | 1,185 | 0,000 |
| Pja2 | praja 2, RING-H2 motif containing | 1434383_at | 1,015 | 0,000 | 1,040 | 0,000 |
| Pja2 | praja 2, RING-H2 motif containing | 1452328_s_at | 1,011 | 0,000 | 1,070 | 0,000 |
| Plat | plasminogen activator, tissue | 1415806_at | -1,022 | 0,000 | -1,201 | 0,000 |
| Plat | plasminogen activator, tissue | 1458553_at | -1,167 | 0,347 | -1,214 | 0,000 |
| Ppfia2 | protein tyrosine phosphatase, receptor type, f polypeptide (PTPRF), interacting protein (liprin), alpha 2 | 1456856_at | -1,241 | 0,832 | -1,117 | 0,000 |
| Ppt1 | palmitoyl-protein thioesterase 1 | 1420015_s_at | -1,005 | 0,000 | -1,075 | 0,000 |
| Ppt1 | palmitoyl-protein thioesterase 1 | 1420016_at | 1,021 | 0,000 | 1,083 | 0,000 |
| Ppt1 | palmitoyl-protein thioesterase 1 | 1422467_at | 1,009 | 0,000 | 1,051 | 0,000 |
| Ppt1 | palmitoyl-protein thioesterase 1 | 1422468_at | 1,050 | 0,000 | 1,032 | 0,000 |
| Ppt1 | palmitoyl-protein thioesterase 1 | 1444884_at | -1,154 | 0,730 | -1,198 | 0,267 |
| Prima1 | proline rich membrane anchor 1 | 1425304_s_at | -1,178 | 0,463 | 1,105 | 0,000 |
| Prrt1 | proline-rich transmembrane protein 1 | 1423562_at | 1,008 | 0,000 | 1,002 | 0,000 |
| Prrt1 | proline-rich transmembrane protein 1 | 1423563_at | -1,083 | 0,288 | -1,048 | 0,000 |
| Prss12 | protease, serine, 12 neurotrypsin (motopsin) | 1420388_at | 1,132 | 0,178 | 1,136 | 0,000 |
| Psd3 | pleckstrin and Sec7 domain containing 3 | 1418749_at | -1,108 | 0,496 | -1,022 | 0,000 |
| Psd3 | pleckstrin and Sec7 domain containing 3 | 1430880_at | -1,052 | 0,000 | 1,057 | 0,000 |
| Pvrl1 | poliovirus receptor-related 1 | 1438111_at | -1,038 | 0,016 | -1,081 | 0,000 |
| Pvrl1 | poliovirus receptor-related 1 | 1438421_at | -1,068 | 0,107 | -1,040 | 0,000 |
| Pvrl1 | poliovirus receptor-related 1 | 1450819_at | -1,094 | 0,092 | -1,040 | 0,000 |
| Rab11b | RAB11B, member RAS oncogene family | 1423448_at | 1,016 | 0,000 | 1,007 | 0,000 |
| Rab11b | RAB11B, member RAS oncogene family | 1435253_at | 1,092 | 0,071 | -1,025 | 0,000 |
| Rab11b | RAB11B, member RAS oncogene family | 1439508_at | 1,036 | 0,000 | 1,007 | 0,000 |
| Rabac1 | Rab acceptor 1 (prenylated) | 1427773_a_at | 1,046 | 0,000 | 1,025 | 0,000 |
| Rapgef2 | Rap guanine nucleotide exchange factor (GEF) 2 | 1452833_at | -1,005 | 0,000 | -1,011 | 0,000 |
| Rapsn | receptor-associated protein of the synapse | 1449331_a_at | -1,033 | 0,000 | -1,103 | 0,000 |
| Rasgrp2 | RAS, guanyl releasing protein 2 | 1417804_at | 1,065 | 0,000 | 1,065 | 0,000 |
| Rasgrp2 | RAS, guanyl releasing protein 2 | 1438932_at | 1,049 | 0,000 | 1,197 | 0,000 |
| Rasgrp2 | RAS, guanyl releasing protein 2 | 1438933_x_at | 1,020 | 0,000 | 1,240 | 0,000 |
| Rasgrp2 | RAS, guanyl releasing protein 2 | 1442264_at | 1,264 | 0,346 | 1,040 | 0,000 |
| Rgs14 | regulator of G-protein signaling 14 | 1419221_a_at | -1,041 | 0,014 | 1,037 | 0,000 |
| Rimbp2 | RIMS binding protein 2 | 1441625_at | -1,155 | 0,673 | -1,089 | 0,000 |
| Rims1 | regulating synaptic membrane exocytosis 1 | 1435667_at | -1,009 | 0,000 | 1,040 | 0,000 |
| Rims1 | regulating synaptic membrane exocytosis 1 | 1438305_at | -1,028 | 0,011 | -1,013 | 0,000 |
| Rims2 | regulating synaptic membrane exocytosis 2 | 1422809_at | 1,014 | 0,000 | -1,002 | 0,000 |
| Rims2 | regulating synaptic membrane exocytosis 2 | 1432102_at | 1,073 | 0,000 | 1,048 | 0,000 |
| Rims2 | regulating synaptic membrane exocytosis 2 | 1436470_at | -1,042 | 0,032 | -1,023 | 0,000 |
| Rims2 | regulating synaptic membrane exocytosis 2 | 1443215_at | -1,057 | 0,000 | -1,035 | 0,000 |
| Rims2 | regulating synaptic membrane exocytosis 2 | 1450761_s_at | -1,063 | 0,090 | 1,005 | 0,000 |
| Rims3 | regulating synaptic membrane exocytosis 3 | 1435971_at | 1,127 | 0,010 | -1,053 | 0,000 |
| Rims3 | regulating synaptic membrane exocytosis 3 | 1459042_at | 1,003 | 0,000 | -1,060 | 0,000 |
| Rims4 | regulating synaptic membrane exocytosis 4 | 1456926_at | 1,011 | 0,000 | 1,097 | 0,000 |
| Rph3a | rabphilin 3A | 1434635_at | -1,024 | 0,000 | -1,013 | 0,000 |
| Rph3a | rabphilin 3A | 1449961_at | -1,081 | 0,165 | 1,091 | 0,000 |
| Rps6kb1 | ribosomal protein S6 kinase, polypeptide 1 | 1428849_at | 1,014 | 0,000 | -1,004 | 0,000 |
| Rps6kb1 | ribosomal protein S6 kinase, polypeptide 1 | 1446376_at | -1,041 | 0,000 | 1,038 | 0,000 |
| Rps6kb1 | ribosomal protein S6 kinase, polypeptide 1 | 1454956_at | 1,036 | 0,000 | 1,120 | 0,000 |
| Rps6kb1 | ribosomal protein S6 kinase, polypeptide 1 | 1457562_at | 1,212 | 0,330 | 1,014 | 0,000 |
| Rps6kb1 | ribosomal protein S6 kinase, polypeptide 1 | 1459951_at | 1,301 | 0,094 | 1,232 | 0,000 |
| Rps6kb1 | ribosomal protein S6 kinase, polypeptide 1 | 1460705_at | 1,042 | 0,000 | 1,133 | 0,000 |
| Rusc1 | RUN and SH3 domain containing 1 | 1431137_at | 1,096 | 0,009 | 1,087 | 0,000 |
| Rusc1 | RUN and SH3 domain containing 1 | 1434743_x_at | -1,045 | 0,040 | 1,014 | 0,000 |
| Rusc1 | RUN and SH3 domain containing 1 | 1436014_a_at | -1,029 | 0,000 | -1,038 | 0,000 |
| Rusc1 | RUN and SH3 domain containing 1 | 1437991_x_at | -1,046 | 0,000 | 1,019 | 0,000 |
| Rusc1 | RUN and SH3 domain containing 1 | 1438017_at | 1,000 | 0,000 | 1,022 | 0,000 |
| Samd4 | sterile alpha motif domain containing 4 | 1424594_at | 1,113 | 0,128 | 1,103 | 0,000 |
| Samd4 | sterile alpha motif domain containing 4 | 1429449_at | 1,070 | 0,000 | 1,106 | 0,000 |
| Samd4 | sterile alpha motif domain containing 4 | 1436356_at | -1,012 | 0,000 | 1,146 | 0,000 |
| Samd4 | sterile alpha motif domain containing 4 | 1454382_at | 1,082 | 0,000 | 1,059 | 0,000 |
| Sarm1 | sterile alpha and HEAT/Armadillo motif containing 1 | 1435466_at | -1,050 | 0,039 | 1,043 | 0,000 |
| Sarm1 | sterile alpha and HEAT/Armadillo motif containing 1 | 1445644_at | -1,113 | 0,081 | 1,137 | 0,000 |
| Sarm1 | sterile alpha and HEAT/Armadillo motif containing 1 | 1457450_at | 1,032 | 0,000 | 1,156 | 0,000 |
| Scamp1 | secretory carrier membrane protein 1 | 1426775_s_at | 1,029 | 0,000 | 1,019 | 0,000 |
| Scamp1 | secretory carrier membrane protein 1 | 1453054_at | 1,050 | 0,000 | 1,164 | 0,000 |
| Scamp5 | secretory carrier membrane protein 5 | 1450247_a_at | 1,079 | 0,000 | 1,066 | 0,000 |
| Scamp5 | secretory carrier membrane protein 5 | 1451224_at | 1,005 | 0,000 | 1,044 | 0,000 |
| Sdc2 | syndecan 2 | 1417011_at | 1,032 | 0,000 | -1,117 | 0,000 |
| Sdc2 | syndecan 2 | 1417012_at | 1,166 | 0,310 | -1,093 | 0,000 |
| Sdc2 | syndecan 2 | 1431474_at | 1,036 | 0,000 | 1,115 | 0,000 |
| Sdc2 | syndecan 2 | 1448545_at | 1,127 | 0,231 | -1,065 | 0,000 |
| Sema4c | sema domain, immunoglobulin domain (Ig), transmembrane domain (TM) and short cytoplasmic domain, (semaphorin) 4C | 1433920_at | 1,078 | 0,000 | -1,024 | 0,000 |
| Sema4f | sema domain, immunoglobulin domain (Ig), TM domain, and short cytoplasmic domain | 1419328_at | -1,004 | 0,000 | 1,155 | 0,000 |
| Sema4f | sema domain, immunoglobulin domain (Ig), TM domain, and short cytoplasmic domain | 1439768_x_at | -1,043 | 0,000 | 1,063 | 0,000 |
| Sez6 | seizure related gene 6 | 1420885_a_at | -1,012 | 0,000 | 1,005 | 0,000 |
| Sez6 | seizure related gene 6 | 1427674_a_at | -1,048 | 0,033 | -1,081 | 0,000 |
| Sez6 | seizure related gene 6 | 1459972_x_at | -1,040 | 0,000 | 1,002 | 0,000 |
| Sh3kbp1 | SH3-domain kinase binding protein 1 | 1431592_a_at | 1,150 | 0,195 | 1,183 | 0,000 |
| Sh3kbp1 | SH3-domain kinase binding protein 1 | 1432269_a_at | 1,342 | 0,513 | 1,370 | 0,000 |
| Sh3kbp1 | SH3-domain kinase binding protein 1 | 1456261_at | 1,114 | 0,013 | 1,044 | 0,000 |
| Sh3kbp1 | SH3-domain kinase binding protein 1 | 1460337_at | 1,089 | 0,220 | 1,145 | 0,000 |
| Shank1 | SH3/ankyrin domain gene 1 | 1444240_at | -1,086 | 0,138 | 1,032 | 0,000 |
| Shank2 | SH3/ankyrin domain gene 2 | 1442823_at | 1,073 | 0,003 | 1,113 | 0,000 |
| Shank3 | SH3/ankyrin domain gene 3 | 1419137_at | -1,020 | 0,000 | 1,077 | 0,000 |
| Shc4 | SHC (Src homology 2 domain containing) family, member 4 | 1457118_at | -1,007 | 0,000 | 1,086 | 0,000 |
| Shisa9 | shisa homolog 9 (Xenopus laevis) | 1435424_x_at | -1,059 | 0,091 | -1,052 | 0,000 |
| Shisa9 | shisa homolog 9 (Xenopus laevis) | 1453428_at | -1,249 | 0,505 | -1,186 | 0,000 |
| Shisa9 | shisa homolog 9 (Xenopus laevis) | 1456995_at | -1,101 | 0,365 | -1,014 | 0,000 |
| Sipa1l1 | signal-induced proliferation-associated 1 like 1 | 1434222_at | -1,116 | 0,309 | -1,007 | 0,000 |
| Sipa1l1 | signal-induced proliferation-associated 1 like 1 | 1439755_at | 1,013 | 0,000 | -1,077 | 0,000 |
| Sipa1l1 | signal-induced proliferation-associated 1 like 1 | 1440119_at | -1,208 | 0,486 | -1,082 | 0,000 |
| Sipa1l1 | signal-induced proliferation-associated 1 like 1 | 1440647_at | -1,041 | 0,000 | 1,081 | 0,000 |
| Slc17a5 | solute carrier family 17 (anion/sugar transporter), member 5 | 1429116_at | -1,028 | 0,000 | -1,068 | 0,000 |
| Slc17a6 | solute carrier family 17 (sodium-dependent inorganic phosphate cotransporter), member 6 | 1418610_at | -1,061 | 0,000 | -1,277 | 0,000 |
| Slc17a6 | solute carrier family 17 (sodium-dependent inorganic phosphate cotransporter), member 6 | 1428379_at | -1,007 | 0,000 | -1,182 | 0,000 |
| Slc17a7 | solute carrier family 17 (sodium-dependent inorganic phosphate cotransporter), member 7 | 1428986_at | -1,022 | 0,000 | 1,050 | 0,000 |
| Slc1a2 | solute carrier family 1 (glial high affinity glutamate transporter), member 2 | 1433094_at | -1,095 | 0,097 | 1,069 | 0,000 |
| Slc1a2 | solute carrier family 1 (glial high affinity glutamate transporter), member 2 | 1438194_at | -1,007 | 0,000 | 1,025 | 0,000 |
| Slc1a2 | solute carrier family 1 (glial high affinity glutamate transporter), member 2 | 1439940_at | 1,080 | 0,000 | 1,159 | 0,000 |
| Slc1a2 | solute carrier family 1 (glial high affinity glutamate transporter), member 2 | 1451627_a_at | -1,046 | 0,077 | 1,009 | 0,000 |
| Slc1a2 | solute carrier family 1 (glial high affinity glutamate transporter), member 2 | 1457800_at | 1,160 | 0,000 | 1,137 | 0,000 |
| Slc1a2 | solute carrier family 1 (glial high affinity glutamate transporter), member 2 | 1459014_at | 1,107 | 0,000 | 1,066 | 0,000 |
| Slc1a3 | solute carrier family 1 (glial high affinity glutamate transporter), member 3 | 1426340_at | -1,039 | 0,006 | -1,045 | 0,000 |
| Slc1a3 | solute carrier family 1 (glial high affinity glutamate transporter), member 3 | 1426341_at | 1,068 | 0,000 | 1,038 | 0,000 |
| Slc1a3 | solute carrier family 1 (glial high affinity glutamate transporter), member 3 | 1439072_at | -1,077 | 0,098 | 1,062 | 0,000 |
| Slc1a3 | solute carrier family 1 (glial high affinity glutamate transporter), member 3 | 1440491_at | -1,051 | 0,012 | -1,057 | 0,000 |
| Slc1a3 | solute carrier family 1 (glial high affinity glutamate transporter), member 3 | 1443749_x_at | -1,099 | 0,188 | -1,090 | 0,000 |
| Slc1a3 | solute carrier family 1 (glial high affinity glutamate transporter), member 3 | 1452031_at | -1,011 | 0,000 | -1,018 | 0,000 |
| Slc30a3 | solute carrier family 30 (zinc transporter), member 3 | 1460654_at | 1,066 | 0,000 | 1,083 | 0,000 |
| Slc32a1 | solute carrier family 32 (GABA vesicular transporter), member 1 | 1422756_at | -1,035 | 0,000 | -1,064 | 0,000 |
| Slc32a1 | solute carrier family 32 (GABA vesicular transporter), member 1 | 1447573_at | 1,071 | 0,000 | 1,107 | 0,000 |
| Slc32a1 | solute carrier family 32 (GABA vesicular transporter), member 1 | 1447574_s_at | -1,006 | 0,000 | 1,136 | 0,000 |
| Slc32a1 | solute carrier family 32 (GABA vesicular transporter), member 1 | 1456457_at | -1,006 | 0,000 | 1,030 | 0,000 |
| Slc6a17 | solute carrier family 6 (neurotransmitter transporter), member 17 | 1436137_at | 1,012 | 0,000 | -1,004 | 0,000 |
| Slc6a17 | solute carrier family 6 (neurotransmitter transporter), member 17 | 1458394_at | 1,086 | 0,016 | 1,118 | 0,000 |
| Slc6a17 | solute carrier family 6 (neurotransmitter transporter), member 17 | 1458441_at | 1,019 | 0,000 | -1,078 | 0,000 |
| Snap23 | synaptosomal-associated protein 23 | 1420896_at | -1,026 | 0,000 | 1,021 | 0,000 |
| Snap23 | synaptosomal-associated protein 23 | 1420897_at | 1,062 | 0,000 | -1,095 | 0,000 |
| Snap23 | synaptosomal-associated protein 23 | 1420898_at | 1,149 | 0,000 | 1,049 | 0,000 |
| Snap25 | synaptosomal-associated protein 25 | 1416828_at | -1,006 | 0,000 | -1,031 | 0,000 |
| Snap29 | synaptosomal-associated protein 29 | 1423354_at | 1,007 | 0,000 | 1,034 | 0,000 |
| Snap29 | synaptosomal-associated protein 29 | 1423355_at | 1,060 | 0,000 | 1,178 | 0,000 |
| Snap29 | synaptosomal-associated protein 29 | 1423356_at | 1,078 | 0,051 | 1,074 | 0,000 |
| Snapin | SNAP-associated protein | 1415756_a_at | -1,062 | 0,161 | 1,018 | 0,000 |
| Snapin | SNAP-associated protein | 1426578_s_at | -1,047 | 0,143 | -1,055 | 0,000 |
| Snca | synuclein, alpha | 1418493_a_at | -2,266 | 0,179 | 2,699 | 0,000 |
| Snca | synuclein, alpha | 1431022_at | -1,133 | 0,232 | -1,047 | 0,000 |
| Snca | synuclein, alpha | 1436853_a_at | -2,165 | 0,179 | 2,420 | 0,000 |
| Sncb | synuclein, beta | 1418053_at | 1,049 | 0,000 | 1,022 | 0,000 |
| Snph | syntaphilin | 1435210_s_at | -1,037 | 0,070 | -1,009 | 0,000 |
| Sntb1 | syntrophin, basic 1 | 1421455_at | 1,135 | 0,127 | 1,191 | 0,000 |
| Sntb1 | syntrophin, basic 1 | 1451938_a_at | 1,014 | 0,000 | -1,096 | 0,000 |
| Sntb2 | syntrophin, basic 2 | 1420371_at | -1,079 | 0,038 | -1,134 | 0,000 |
| Sntb2 | syntrophin, basic 2 | 1420372_at | -1,322 | 0,666 | -1,200 | 0,000 |
| Sntb2 | syntrophin, basic 2 | 1436986_at | -1,114 | 0,086 | -1,431 | 0,000 |
| Sntb2 | syntrophin, basic 2 | 1449840_at | -1,037 | 0,000 | -1,034 | 0,000 |
| Sparcl1 | SPARC-like 1 | 1416114_at | -1,077 | 0,301 | -1,081 | 0,000 |
| Spg20 | spastic paraplegia 20, spartin (Troyer syndrome) homolog (human) | 1424875_at | 1,097 | 0,029 | 1,034 | 0,000 |
| Spg20 | spastic paraplegia 20, spartin (Troyer syndrome) homolog (human) | 1424876_s_at | 1,075 | 0,000 | 1,098 | 0,000 |
| Spg20 | spastic paraplegia 20, spartin (Troyer syndrome) homolog (human) | 1451520_at | -1,030 | 0,000 | 1,007 | 0,000 |
| Srcin1 | SRC kinase signaling inhibitor 1 | 1420706_at | 1,074 | 0,042 | 1,169 | 0,000 |
| Srcin1 | SRC kinase signaling inhibitor 1 | 1436621_at | 1,038 | 0,000 | 1,095 | 0,000 |
| Srgap2 | SLIT-ROBO Rho GTPase activating protein 2 | 1429884_at | 1,017 | 0,000 | 1,004 | 0,000 |
| Srgap2 | SLIT-ROBO Rho GTPase activating protein 2 | 1434406_at | 1,054 | 0,122 | 1,030 | 0,000 |
| Srgap2 | SLIT-ROBO Rho GTPase activating protein 2 | 1434407_at | 1,113 | 0,225 | 1,056 | 0,000 |
| Sspn | sarcospan | 1417644_at | 1,113 | 0,051 | -1,041 | 0,000 |
| Sspn | sarcospan | 1417645_at | 1,142 | 0,294 | 1,132 | 0,000 |
| Stx1a | syntaxin 1A (brain) | 1437390_x_at | 1,035 | 0,000 | 1,152 | 0,000 |
| Stx1a | syntaxin 1A (brain) | 1448366_at | 1,209 | 0,649 | 1,223 | 0,000 |
| Sumo1 | SMT3 suppressor of mif two 3 homolog 1 (yeast) | 1438289_a_at | -1,016 | 0,000 | -1,004 | 0,000 |
| Sumo1 | SMT3 suppressor of mif two 3 homolog 1 (yeast) | 1451005_at | 1,007 | 0,000 | -1,036 | 0,000 |
| Sumo1 | SMT3 suppressor of mif two 3 homolog 1 (yeast) | 1456349_x_at | -1,005 | 0,000 | -1,019 | 0,000 |
| Sv2a | synaptic vesicle glycoprotein 2 a | 1423406_at | 1,037 | 0,000 | -1,031 | 0,000 |
| Sv2b | synaptic vesicle glycoprotein 2 b | 1434800_at | 1,040 | 0,000 | 1,044 | 0,000 |
| Sv2b | synaptic vesicle glycoprotein 2 b | 1435687_at | 1,019 | 0,000 | 1,170 | 0,104 |
| Sv2c | synaptic vesicle glycoprotein 2c | 1453715_at | 1,091 | 0,000 | -1,042 | 0,000 |
| Svop | SV2 related protein | 1452663_at | -1,014 | 0,000 | 1,088 | 0,000 |
| Syn1 | synapsin I | 1451484_a_at | -1,071 | 0,157 | -1,015 | 0,000 |
| Syn2 | synapsin II | 1428460_at | 1,020 | 0,000 | -1,018 | 0,000 |
| Syn2 | synapsin II | 1435511_at | -1,165 | 0,451 | -1,095 | 0,000 |
| Syn2 | synapsin II | 1440762_at | -1,447 | 0,987 | 1,034 | 0,000 |
| Syn2 | synapsin II | 1449030_at | -1,055 | 0,094 | 1,024 | 0,000 |
| Syn2 | synapsin II | 1458105_at | -1,128 | 0,092 | -1,052 | 0,000 |
| Syn2 | synapsin II | 1460230_at | -1,056 | 0,178 | -1,006 | 0,000 |
| Syn3 | synapsin III | 1421248_at | -1,010 | 0,000 | 1,067 | 0,000 |
| Syn3 | synapsin III | 1435310_at | 1,096 | 0,000 | -1,140 | 0,000 |
| Syn3 | synapsin III | 1435311_s_at | 1,018 | 0,000 | -1,145 | 0,000 |
| Syngr1 | synaptogyrin 1 | 1419289_a_at | 1,105 | 0,261 | 1,197 | 0,081 |
| Syngr1 | synaptogyrin 1 | 1434661_at | 1,013 | 0,000 | 1,046 | 0,000 |
| Syngr1 | synaptogyrin 1 | 1453772_at | -1,002 | 0,000 | 1,018 | 0,000 |
| Syngr3 | synaptogyrin 3 | 1416098_at | -1,021 | 0,000 | 1,013 | 0,000 |
| Syngr3 | synaptogyrin 3 | 1448216_at | 1,099 | 0,206 | 1,119 | 0,000 |
| Synpo | synaptopodin | 1427045_at | -1,115 | 0,403 | -1,036 | 0,000 |
| Synpo | synaptopodin | 1434089_at | -1,135 | 0,176 | -1,236 | 0,000 |
| Synpr | synaptoporin | 1423640_at | -1,046 | 0,089 | -1,001 | 0,000 |
| Syp | synaptophysin | 1448280_at | 1,026 | 0,000 | 1,026 | 0,000 |
| Syp | synaptophysin | 1456249_x_at | 1,068 | 0,056 | 1,058 | 0,000 |
| Syt1 | synaptotagmin I | 1421990_at | -1,022 | 0,000 | 1,086 | 0,000 |
| Syt1 | synaptotagmin I | 1431191_a_at | -1,038 | 0,000 | 1,097 | 0,000 |
| Syt1 | synaptotagmin I | 1433884_at | -1,013 | 0,000 | -1,019 | 0,000 |
| Syt1 | synaptotagmin I | 1438282_at | -1,195 | 0,616 | -1,030 | 0,000 |
| Syt10 | synaptotagmin X | 1450347_at | -1,063 | 0,043 | -1,071 | 0,000 |
| Syt11 | synaptotagmin XI | 1429314_at | -1,040 | 0,091 | 1,046 | 0,000 |
| Syt11 | synaptotagmin XI | 1429315_at | -1,045 | 0,162 | 1,004 | 0,000 |
| Syt11 | synaptotagmin XI | 1449264_at | 1,046 | 0,000 | 1,145 | 0,000 |
| Syt11 | synaptotagmin XI | 1455176_a_at | -1,016 | 0,000 | -1,004 | 0,000 |
| Syt11 | synaptotagmin XI | 1456464_x_at | -1,046 | 0,000 | 1,046 | 0,000 |
| Syt12 | synaptotagmin XII | 1422878_at | 1,021 | 0,000 | 1,011 | 0,000 |
| Syt2 | synaptotagmin II | 1420418_at | -1,289 | 0,447 | -1,148 | 0,000 |
| Syt2 | synaptotagmin II | 1440323_at | -1,057 | 0,026 | -1,185 | 0,000 |
| Syt2 | synaptotagmin II | 1449866_at | -1,068 | 0,031 | 1,031 | 0,000 |
| Syt3 | synaptotagmin III | 1417708_at | 1,037 | 0,000 | 1,121 | 0,000 |
| Syt4 | synaptotagmin IV | 1415844_at | -1,075 | 0,138 | -1,108 | 0,000 |
| Syt4 | synaptotagmin IV | 1415845_at | -1,044 | 0,092 | -1,040 | 0,000 |
| Syt5 | synaptotagmin V | 1422531_at | 1,058 | 0,000 | 1,027 | 0,000 |
| Syt6 | synaptotagmin VI | 1420188_at | -1,005 | 0,000 | 1,109 | 0,000 |
| Syt6 | synaptotagmin VI | 1426106_a_at | -1,032 | 0,000 | 1,066 | 0,000 |
| Syt6 | synaptotagmin VI | 1449766_at | -1,014 | 0,000 | 1,151 | 0,000 |
| Syt6 | synaptotagmin VI | 1449767_x_at | -1,016 | 0,000 | 1,034 | 0,000 |
| Syt7 | synaptotagmin VII | 1423012_at | -1,220 | 0,581 | -1,020 | 0,000 |
| Syt7 | synaptotagmin VII | 1439633_at | -1,266 | 0,975 | -1,208 | 0,314 |
| Syt7 | synaptotagmin VII | 1441927_at | -1,215 | 0,750 | -1,068 | 0,000 |
| Syt7 | synaptotagmin VII | 1460081_at | -1,336 | 0,993 | -1,138 | 0,000 |
| Syt9 | synaptotagmin IX | 1423258_at | 1,259 | 0,327 | -1,436 | 0,000 |
| Tanc1 | tetratricopeptide repeat, ankyrin repeat and coiled-coil containing 1 | 1452714_at | -1,126 | 0,484 | -1,092 | 0,000 |
| Tln2 | talin 2 | 1429111_at | -1,139 | 0,287 | 1,108 | 0,000 |
| Tln2 | talin 2 | 1429112_at | 1,061 | 0,000 | 1,261 | 0,000 |
| Tln2 | talin 2 | 1435700_at | -1,078 | 0,198 | 1,041 | 0,000 |
| Tmem57 | transmembrane protein 57 | 1428883_at | -1,009 | 0,000 | -1,066 | 0,000 |
| Tmem57 | transmembrane protein 57 | 1428884_at | -1,038 | 0,045 | -1,092 | 0,000 |
| Tmem57 | transmembrane protein 57 | 1430418_at | 1,026 | 0,000 | -1,067 | 0,000 |
| Tmem57 | transmembrane protein 57 | 1458545_at | -1,042 | 0,024 | -1,016 | 0,000 |
| Tprgl | transformation related protein 63 regulated like | 1451172_at | -1,069 | 0,265 | -1,067 | 0,000 |
| Trappc4 | trafficking protein particle complex 4 | 1415674_a_at | 1,090 | 0,270 | 1,092 | 0,000 |
| Trappc4 | trafficking protein particle complex 4 | 1429632_at | 1,126 | 0,052 | -1,053 | 0,000 |
| Trim9 | tripartite motif-containing 9 | 1434249_s_at | -1,010 | 0,000 | -1,003 | 0,000 |
| Trim9 | tripartite motif-containing 9 | 1434595_at | 1,003 | 0,000 | 1,015 | 0,000 |
| Trim9 | tripartite motif-containing 9 | 1443989_at | 1,022 | 0,000 | -1,081 | 0,000 |
| Trim9 | tripartite motif-containing 9 | 1454886_x_at | -1,059 | 0,194 | -1,007 | 0,000 |
| Trpv1 | transient receptor potential cation channel, subfamily V, member 1 | 1443392_at | -1,125 | 0,328 | -1,023 | 0,000 |
| Tulp1 | tubby like protein 1 | 1451582_at | -1,056 | 0,000 | 1,069 | 0,000 |
| Ube2i | ubiquitin-conjugating enzyme E2I | 1422713_a_at | 1,032 | 0,000 | 1,114 | 0,000 |
| Ube2i | ubiquitin-conjugating enzyme E2I | 1425478_x_at | -1,025 | 0,000 | 1,014 | 0,000 |
| Ube2i | ubiquitin-conjugating enzyme E2I | 1453189_at | 1,211 | 0,235 | 1,018 | 0,000 |
| Unc13a | unc-13 homolog A (C. elegans) | 1437472_at | -1,045 | 0,090 | 1,079 | 0,000 |
| Unc13b | unc-13 homolog B (C. elegans) | 1417757_at | -1,151 | 0,424 | -1,012 | 0,000 |
| Unc13b | unc-13 homolog B (C. elegans) | 1443308_at | -1,009 | 0,000 | 1,194 | 0,000 |
| Unc13c | unc-13 homolog C (C. elegans) | 1430511_at | -1,087 | 0,034 | -1,060 | 0,000 |
| Unc13c | unc-13 homolog C (C. elegans) | 1437319_at | -1,110 | 0,090 | -1,074 | 0,000 |
| Unc13c | unc-13 homolog C (C. elegans) | 1455304_at | -1,165 | 0,174 | -1,106 | 0,000 |
| Ush1c | Usher syndrome 1C homolog (human) | 1450001_a_at | -1,170 | 0,434 | -1,071 | 0,000 |
| Usp14 | ubiquitin specific peptidase 14 | 1416208_at | 1,122 | 0,477 | 1,038 | 0,000 |
| Usp14 | ubiquitin specific peptidase 14 | 1437714_x_at | -1,002 | 0,000 | 1,017 | 0,000 |
| Usp14 | ubiquitin specific peptidase 14 | 1439201_at | -1,055 | 0,073 | 1,064 | 0,000 |
| Usp14 | ubiquitin specific peptidase 14 | 1455829_at | 1,018 | 0,000 | 1,057 | 0,000 |
| Utrn | utrophin | 1426892_at | 1,166 | 0,000 | 1,276 | 0,000 |
| Utrn | utrophin | 1427569_a_at | -1,018 | 0,000 | -1,015 | 0,000 |
| Utrn | utrophin | 1452222_at | 1,047 | 0,000 | -1,019 | 0,000 |
| Vamp1 | vesicle-associated membrane protein 1 | 1421862_a_at | 1,080 | 0,000 | 1,103 | 0,000 |
| Vamp1 | vesicle-associated membrane protein 1 | 1421863_at | -1,080 | 0,074 | 1,033 | 0,000 |
| Vamp2 | vesicle-associated membrane protein 2 | 1420833_at | 1,153 | 0,176 | 1,150 | 0,000 |
| Vamp2 | vesicle-associated membrane protein 2 | 1420834_at | 1,017 | 0,000 | -1,036 | 0,000 |
| Vamp3 | vesicle-associated membrane protein 3 | 1421102_a_at | 1,271 | 0,438 | 1,092 | 0,000 |
| Vamp3 | vesicle-associated membrane protein 3 | 1433693_x_at | 1,092 | 0,000 | 1,068 | 0,000 |
| Vamp3 | vesicle-associated membrane protein 3 | 1433916_at | 1,142 | 0,211 | -1,005 | 0,000 |
| Vamp3 | vesicle-associated membrane protein 3 | 1437708_x_at | 1,135 | 0,010 | 1,010 | 0,000 |
| Vamp3 | vesicle-associated membrane protein 3 | 1456245_x_at | 1,077 | 0,000 | 1,008 | 0,000 |
| Vamp3 | vesicle-associated membrane protein 3 | 1457391_at | -1,029 | 0,000 | -1,212 | 0,000 |
| Vamp7 | vesicle-associated membrane protein 7 | 1426269_at | -1,005 | 0,000 | 1,032 | 0,000 |
| Vamp7 | vesicle-associated membrane protein 7 | 1452007_at | 1,020 | 0,000 | -1,012 | 0,000 |
| Vwc2 | von Willebrand factor C domain containing 2 | 1441427_at | -1,055 | 0,010 | 1,245 | 0,000 |
| Vwc2 | von Willebrand factor C domain containing 2 | 1455882_x_at | 1,054 | 0,000 | -1,059 | 0,000 |
| Vwc2l | von Willebrand factor C domain-containing protein 2-like | 1440413_at | -1,216 | 0,091 | -1,101 | 0,000 |
| Whrn | whirlin | 1432555_at | 1,262 | 0,442 | 1,019 | 0,000 |
| Whrn | whirlin | 1436485_s_at | 1,355 | 0,159 | 1,117 | 0,000 |
| Whrn | whirlin | 1436486_x_at | -1,024 | 0,000 | -1,161 | 0,000 |
| Whrn | whirlin | 1442507_at | 1,039 | 0,000 | -1,181 | 0,000 |
| Zc4h2 | zinc finger, C4H2 domain containing | 1434729_at | 1,048 | 0,000 | 1,093 | 0,000 |
| Zmynd19 | zinc finger, MYND domain containing 19 | 1419522_at | -1,043 | 0,045 | -1,140 | 0,000 |
| Zmynd19 | zinc finger, MYND domain containing 19 | 1440205_at | -1,016 | 0,000 | 1,111 | 0,000 |
| Zmynd19 | zinc finger, MYND domain containing 19 | 1447403_a_at | 1,013 | 0,000 | -1,015 | 0,000 |
| Znrf1 | zinc and ring finger 1 | 1420949_at | 1,037 | 0,000 | 1,031 | 0,000 |
| Znrf1 | zinc and ring finger 1 | 1420950_at | -1,051 | 0,104 | -1,034 | 0,000 |
| Znrf1 | zinc and ring finger 1 | 1424384_a_at | -1,016 | 0,000 | -1,017 | 0,000 |
| Znrf2 | zinc and ring finger 2 | 1431154_at | -1,037 | 0,000 | -1,045 | 0,000 |
| Znrf2 | zinc and ring finger 2 | 1434016_at | 1,064 | 0,000 | 1,116 | 0,000 |
| Znrf2 | zinc and ring finger 2 | 1434017_at | 1,075 | 0,153 | 1,043 | 0,000 |
| Znrf2 | zinc and ring finger 2 | 1455290_at | -1,034 | 0,042 | 1,022 | 0,000 |
| Znrf2 | zinc and ring finger 2 | 1455291_s_at | 1,008 | 0,000 | 1,024 | 0,000 |
