# Supplementary material for: Reelin Exerts Structural, Biochemical and Transcriptional Regulation Over Presynaptic and Postsynaptic Elements in the Adult Hippocampus
Source: Front Cell Neurosci. 2016 May 30;10:138. doi: 10.3389/fncel.2016.00138 (PMC4884741; doi:10.3389/fncel.2016.00138)
Supplement: TABLE S2 — Gene expression of selected genes. The fold-change (fc) and the probability (prob) of differential expression are presented for all the probes in the Genechip Mouse Genome 430 2.0 array corresponding to Limk1/Limk2/Cfl1/Clf2 genes. Reelin-OE mice (R-OE) and Reelin-OE mice treated for one week with doxycycline (R-OE+1w(DOX)) were compared with controls. [file Table_2.DOCX]

| **Symbol** | **Gene name** | **Accession** | **Control - Reelin-OE** | | **Control - Reelin-OE-DOX** | |
| --- | --- | --- | --- | --- | --- | --- |
|  |  |  | **Fold change** | **P** | **Fold change** | **P** |
| Cfl1 | cofilin 1, non-muscle | 1455138_x_at | -1,043 | 0,091 | -1,051 | 0,000 |
| Cfl1 | cofilin 1, non-muscle | 1448346_at | 1,083 | 0,074 | 1,147 | 0,000 |
| Cfl2 | cofilin 2, muscle | 1431432_at | -1,123 | 0,335 | 1,021 | 0,000 |
| Cfl2 | cofilin 2, muscle | 1418067_at | 1,031 | 0,000 | -1,069 | 0,000 |
| Cfl2 | cofilin 2, muscle | 1418066_at | 1,070 | 0,000 | -1,128 | 0,000 |
| Limk1 | LIM-domain containing, protein kinase | 1417627_a_at | 1,013 | 0,000 | 1,074 | 0,000 |
| Limk1 | LIM-domain containing, protein kinase | 1425836_a_at | -1,020 | 0,000 | 1,034 | 0,000 |
| Limk1 | LIM-domain containing, protein kinase | 1456234_at | -1,046 | 0,000 | -1,065 | 0,000 |
| Limk2 | LIM motif-containing protein kinase 2 | 1452060_a_at | -1,041 | 0,022 | -1,039 | 0,000 |
| Limk2 | LIM motif-containing protein kinase 2 | 1418581_a_at | 1,001 | 0,000 | 1,046 | 0,000 |
| Limk2 | LIM motif-containing protein kinase 2 | 1439896_at | 1,001 | 0,000 | -1,070 | 0,000 |
